# Preparation and bioactivity of probiotic-fermented lotus seed and lily bulb beverage

**DOI:** 10.3389/fmicb.2026.1796122

**Published:** 2026-05-14

**Authors:** Xiaoqing Xu, Yifan Wang, Lingshan Liang, Chuncheng Wang, Qingfen Xie, Bei Ni, Jinmin Liang, Xiaotong Liao, Yuan Xie, Quliang Gu, He Li

**Affiliations:** 1 School of Base Medical Sciences, Guangdong Pharmaceutical University, Guangzhou, China; 2 College of Grassland Science, Qingdao Agricultural University, Qingdao, China

**Keywords:** biological activity, fermentation, lotus seed lily, microbial, probiotics

## Abstract

**Introduction:**

Lotus seeds and lily bulbs are rich in active components, including polysaccharides, flavonoids, polyphenols, and saponins, which exhibit a range of biological activities such as antioxidant, anti-inflammatory, and immune-regulating properties. Consequently, lotus seeds and lily bulbs find extensive applications in the food and cosmetics industries.

**Method:**

First, lotus seeds, lily bulbs, and their mixtures were fermented using 19 probiotic strains including Saccharomyces and Lactobacillus. The fermentation methods and strains were screened by comparing the total polysaccharide and total flavonoid contents in the fermentation products under different strains and fermentation methods. Second, single-factor and response surface optimization methods were employed. Using Design-Expert 10 software for Box-Behnken experimental design, with free radical scavenging rate as the response value, three factors affecting the polysaccharide content of the lotus seed and lily bulb fermentation broth were investigated: fermentation time, material concentration, and temperature. Third, the safety of the fermentation products under optimized conditions was evaluated using the chicken embryo chorioallantois membrane as an alternative model for *in vitro* eye irritation testing. Fourthly, LPS-induced mouse macrophage cell line RAW264.7 monocytes were used as a cellular inflammation model, H_2_O_2_-induced human immortalized keratinocytes (HACAT) as a cellular oxidation model, and mouse melanoma cells B16-F10 to evaluate the *in vitro* anti-inflammatory, antioxidant, and melanin-inhibiting effects of the fermented products. H_2_O_2_-induced zebrafish embryos were employed as an animal oxidation model, copper sulfate-induced zebrafish as an animal inflammation model, and zebrafish larvae to assess the *in vivo* anti-inflammatory, antioxidant, melanin-inhibiting, and skin-whitening effects of the fermented products. Additionally, the TCC method and K-B disk diffusion method were used to test the inhibitory effects of the fermented products against common acne-causing pathogens such as Staphylococcus aureus, Propionibacterium acnes, and Staphylococcus epidermidis.

**Result:**

*Lactobacillus plantarum* L-08 was selected as the dominant strain for fermentation from a pool of 20 probiotics. The optimized fermentation conditions were established as follows: fermentation temperature of 28°C, a feed-to-liquid ratio of 1:35, fermentation time of 48 h, and an inoculation rate of 1%. Probiotic fermentation significantly enhanced the antioxidant and anti-inflammatory biological activities of the lotus seed lily mixed fermentation broth. In cellular experiments, the safe concentration range of the lotus seed lily fermentation broth for various cell types was determined to be between 0.625 and 10%. In the HACAT cell oxidation model, the lotus seed lily fermentation broth significantly increased total antioxidant capacity. In the RAW 264.7 macrophage model, it effectively inhibited the production of inflammatory mediators. In the B16-F10 melanoma cell model, it inhibited melanin synthesis pathways, demonstrating significant whitening effects. In zebrafish injury experiments, the lotus seed lily mixed fermentation broth significantly improved the total antioxidant capacity of zebrafish within the concentration range of 2.5–10%, while also exhibiting anti-inflammatory effects. In chicken embryo chorioallantois membrane experiments, the lotus seed lily mixed fermentation broth did not induce significant damage to the chorioallantoic membrane vessels, thereby proving its safety. A comparative analysis between unfermented group and probiotic fermented group verified that probiotic fermentation significantly enhanced the antioxidant and anti-inflammatory biological activities of the lotus seed lily mixed broth.

**Discussion:**

Lotus seed and lily hybrid fermentation liquid has antioxidant and anti-inflammatory biological activity, and has exhibited low cytotoxicity with cell viability above 90%, which can expand its application in health care, cosmeceutical application.

## 1 Introduction

Lotus seeds, derived from the dried mature seeds of the plant *Nymphaea* within the family *Nymphaeaceae*, are not only palatable but also nutritionally rich. They contain active components such as polysaccharides, flavonoids, polyphenols, and saponins, which exhibit various biological activities, including anti-inflammatory (Bellahcene et al., 2026), antioxidant, and immune-regulating effects. Similarly, lily, a member of the family Liliaceae, is widely utilized for both culinary and medicinal purposes. Clinically, it is regarded as a valuable herb for nourishing yin, tonifying the lungs, clearing heat, and calming the mind. Rich in polysaccharides, flavonoids, and saponins, lily possesses pharmacological effects that include antibacterial, antioxidant, anti-inflammatory, and immune regulation (Abbate et al., 2021). The effective components and pharmacological actions of lotus seeds and lily are subjects of significant interest among scholars both domestically and internationally. In the medical domain, these plants are frequently employed to inhibit inflammatory responses, regulate blood glucose levels, and reduce lipid content. Furthermore, both lotus seeds and lily exhibit potential in preventing skin oxidation, making them suitable for incorporation into anti-wrinkle and skin health-enhancing cosmetic formulations, thereby indicating their promising applications within the cosmetics industry.

Microbial fermentation technology, as a biocatalytic process that utilizes microbial metabolic activities to convert substrates. The mechanism of microbial fermentation mainly includes the following aspects: First, microorganisms secrete extracellular enzymes to biodegrade the cell walls of organic substrates, enhancing the permeability of the cell membrane and fully releasing and utilizing the nutrients of the substrate. Second, secondary metabolites produced by microbial metabolic activities can alter the composition of substrates through structural modifications, thereby affecting their properties and functions (Papagianni, 2004). Compared to chemical methods, microbial fermentation technology offers better controllability and higher safety, with fewer by-products. Therefore, this technology has broad application prospects in the food industry, pharmaceutical production, bioenergy production, and environmental protection.

Production of probiotics is a systematic process integrating microbiology, fermentation engineering, and food processing technology. Its core lies in culturing specific beneficial microorganisms, which are then processed into products suitable for preservation and consumption. The main production steps are as follows: Strain Screening and Cultivation, Strain Selection: First, strains with high activity, good stability, and beneficial effects on the human body. Are screened from natural environments. These strains must pass safety and functional tests, including acid resistance, colonization ability, and probiotic efficacy. Pure Culture: The selected high-quality strains are subjected to pure culture in the laboratory to ensure no contamination by miscellaneous bacteria, that are typically stored in low-temperature or freeze-dried environments to maintain their activity. Large-Scale Fermentation Culture, Seed Scale-Up: The preserved seed cultures are gradually expanded, transferred from laboratory-scale shake flasks to pilot-scale fermenters, and finally to industrial-scale large fermenters to rapidly increase the bacterial population. Fermentation Production: In large fermenters, conditions such as temperature, pH, and oxygen content are controlled. Carbon sources, nitrogen sources, vitamins, and other nutrients are provided to allow the probiotics to multiply in large quantities, reaching the target concentration. Harvest and Concentration. After fermentation, probiotic cells are separated from the fermentation broth using separation technologies such as centrifugation and filtration. Waste liquid and impurities are removed to obtain concentrated bacterial sludge. Freeze-Drying or Inactivation. Viable Probiotic Products: To extend shelf life, the bacterial sludge is subjected to freeze-drying, maximizing the retention of viable bacterial activity. *Lactobacillus plantarum* L-08 is a superior probiotic strain employed in this study. It possesses strong adaptability to the herbal matrix of lotus seed and lily bulb, and can efficiently mediate the biotransformation of macromolecules into bioactive small-molecule metabolites. The fermentation by L-08 significantly elevates the content of skin-functional components, endowing the product with enhanced antioxidant, anti-aging, and skin-repairing activities for cosmetic and aesthetic applications.

The purpose of this study is to prepare fermented lotus seed and lily through probiotic fermentation, and investigate its feasibility for health maintenance, beauty, and pharmaceutical applications. The study involved preparing 19 types of fermented lotus seed and lily using probiotics, comparing the total flavonoid content and total polysaccharide content in each group, to comprehensively screen out the most suitable strains for lotus seed and lily fermentation.

## 2 Materials and methods

### 2.1 Materials and reagents

Lotus seeds and lily bulbs: all purchased from the Anhui Bozhou Chinese Medicine Trading Center. The lactic acid bacteria and yeast used in this experiment are both preserved at the Biochemistry and Molecular Biology Laboratory of Guangdong Pharmaceutical University. The Total Antioxidant Capacity (T-AOC) kit, Malondialdehyde (MDA) kit, and Total Superoxide Dismutase (T-SOD) kit were purchased from Nanjing Jiancheng Institute of Biotechnology. DMEM high-glucose medium, fetal bovine serum, trypsin, superoxide dismutase (SOD) kit, and RPMI-1640 medium were all purchased from Gibco Inc. Human immortal keratinocytes (HACAT), mouse melanoma cells (B16-F10), and mouse mononuclear macrophages (RAW246.7) are self-cold-stored at the Biochemistry and Molecular Biology Laboratory of Guangdong Pharmaceutical University. The AB strain of zebrafish was purchased from the National Aquatic Germplasm Resource Bank Zebrafish Resource Center (CZRC) and hatched and bred in our laboratory. The transgenic zebrafish Tg (corola: EGFP), which marks mature green fluorescent inflammatory cells, was introduced by Guangdong Pharmaceutical University and propagated in our laboratory.

### 2.2 Optimization of lotus seed and lily fermentation process

#### 2.2.1 Activation of lotus seed and lily fermentation strains

The probiotics used in the experiment consisted of lactic acid bacteria and yeasts. Lactic acid bacteria included strains: L-01, L-05, B-01, L-07, L-06, P-01, L-02, L-08, L-04, L-09, L-03, L-10. Yeasts included strains: Y-08, Y-12, Y-13, Y-14, Y-15, Y-16, Y-18. A total of 19 strains were used.

##### 2.2.1.1 Lactic acid bacteria

Lactic acid bacteria were taken out from the ultra-low temperature refrigerator, thawed at 37°C, inoculated into MRS liquid medium, and cultured in a constant temperature shaker (37°C,160 rpm) for 6 h. The absorbance value at 630 nm was measured by a microplate reader to keep the absorbance value within the range of 0.8–1.2 (Ayivi et al., 2022).

##### 2.2.1.2 Yeast

Yeast was taken out from the ultra-low temperature refrigerator, thawed at 37°C and inoculated into PDA liquid medium. It was cultured in a constant temperature shaker (25°C,220 rpm) for 6 h. The absorbance value at 630 nm was measured with a microplate reader to keep the absorbance value within the range of 0.8–1.2 (Wang et al., 2021).

#### 2.2.2 Preparation of lotus seed and lily fermented liquid

Fresh lotus seeds and lilies were dried in a forced-air drying oven at 60°C, then pulverized using a high-speed universal grinder and sieved through a 60-mesh sieve. The powder was degreased with petroleum ether, autoclaved at 121°C for 20 min, and stored for later use.

Lotus seeds are fermented separately: Take 2 g of lotus seed powder that has undergone autoclaving and transfer it to a conical flask. Add sterile water (60°C, 40 mL) to the flask at a material-to-liquid ratio of 1:20 (g:mL) in a laminar flow cabinet. There are 20 fermentation groups in total. Each fermentation group is supplemented with 1% (v/v) probiotic strain seed solution, while the control group uses sterile water instead of the seed solution. The yeast group ferments for 48 h under conditions of 28°C and 150 rpm in a constant temperature shaker, while the lactic acid bacteria group ferments for 48 h (Walker and Walker, 2018) under conditions of 37°C and 150 rpm in a constant temperature shaker. After fermentation, centrifuge (8,000 rpm, 10 min) to collect the supernatant, which serves as the lotus seed fermentation broth for different strains, and store it at 4°C for later use.

The lily is fermented alone: Take 2 g of lily powder sterilized by high pressure into a conical flask. The subsequent operation steps are the same as the separate fermentation of lotus seeds to obtain lily fermentation liquid of different strains, which is stored in 4°C for future use.

Lotus seeds and lilies are fermented together: 1 g lotus seed powder and 1 g lily powder were, respectively, taken to a conical flask after autoclaving, and the subsequent steps were the same as above. The fermented lotus seed and lily fermentation liquid of different strains was obtained and stored in 4°C for later use.

#### 2.2.3 Screening of lotus seed and lily fermentation strains

The polysaccharide content of fermentation broth was used as the index for primary screening, and the flavonoid content was used as the index for secondary screening. The total polysaccharide content in plants is typically determined using the phenol-sulfuric acid method. In a 10 mL centrifuge tube, sequentially add 1 mL of sample, 1 mL of 5% phenol solution, and 5 mL of concentrated sulfuric acid. After settling for 10 min, mix the reaction system and incubate at room temperature for 30 min. Measure the absorbance values of each combination at 490 nm after the reaction. Plot a standard curve with glucose content on the *y*-axis and absorbance values on the *x*-axis. The total flavonoid content in fermentation broth is determined using the aluminum nitrate colorimetric method. The procedure involves adding 1 mL of sample to a 10 mL centrifuge tube, followed by 1 mL of 5% sodium nitrite solution. After mixing and settling for 6 min, add 1 mL of 10% aluminum nitrate solution and mix again, then let it settle for another 6 min. Next, add 4 mL of 4% sodium hydroxide solution and adjust to 10 mL with double-distilled water. React for 15 min. After the reaction, measure the absorbance of the sample at 510 nm and plot a standard curve with rutin content on the *y*-axis and absorbance values on the *x*-axis.

#### 2.2.4 Screening of the mixing fermentation ratio of lotus seeds and lilies

Take the lotus seed powder and lily powder that have undergone high-pressure sterilization, and mix them according to different lily-to-lotus ratios to form the fermentation substrate. Experimental Grouping and Subgroups, Group 1: Raw Material Pretreatment, Subgroup 1: Autoclaving of lotus seed powder, Subgroup 2: Autoclaving of lily bulb powder. Group 2: Mixed Ratio Fermentation Substrate Group, Subgroup 1: Lily bulb : Lotus seed = 0:6, Subgroup 2: Lily bulb : Lotus seed = 1:5, Subgroup 3: Lily bulb : Lotus seed = 2:4, Subgroup 4: Lily bulb : Lotus seed = 3:3, Subgroup 5: Lily bulb : Lotus seed = 4:2, Subgroup 6: Lily bulb : Lotus seed = 5:1, Subgroup 7: Lily bulb : Lotus seed = 6:0. Then, add the mixed powders to a conical flask at a material-to-liquid ratio of 1:20 (g:mL), and add 60°C of sterile water to the flask, cooling it to room temperature. Add a 1% seed solution of dominant strains, where the yeast group is fermented at 28°C and 150 rpm for 48 h in a constant temperature shaker, and the lactic acid bacteria group is fermented at 37°C and 150 rpm for 48 h in a constant temperature shaker. After fermentation, centrifuge separately (8,000 rpm, 10 min) to collect the supernatant, which serves as the lotus seed fermentation broth for different strains.

#### 2.2.5 Response surface method was used to optimize the mixed fermentation process system of lotus seeds and lilies

##### 2.2.5.1 Single factor experiment

The single-factor method was used to study the process conditions of lotus seed and lily hybrid fermentation, with polysaccharide content as the indicator, to investigate the effects of fermentation time, fermentation temperature, feed-to-batch ratio, and inoculum amount on the polysaccharide content in the fermentation broth. The L-08 Lactobacillus strain was used for mixed fermentation of lotus seeds and lilies, with a lily-to-lotus seed ratio of 4:2.

##### 2.2.5.2 Plackett-Burman design

The P-B experiment (Yan et al., 2021) focuses on the impact of different factors on the total polysaccharide content in lotus seed lily fermentation broth. The variable levels selected are based on the values before and after the total polysaccharide content reaches its maximum under each fermentation condition. The experimental design includes different levels of feed-to-batch ratio (low level: 1 g:30 mL, high level: 1 g:35 mL), bacterial inoculation rate (low level: 0.5%, high level: 1%), fermentation time (low level: 36 h, high level: 48 h), and fermentation temperature (low level: 25°C, high level: 28°C). These factors were input into the Design-Expert 10 software for analysis, with the total polysaccharide content in lotus seed lily as the key indicator. Twelve experimental schemes were formulated, as shown in Table 1.

**TABLE 1:** Plackett–Burman experimental design and results.

Run	A	B	C	D	Polysaccharide content mg/g
1	1	1	−1	−1	117.080
2	1	−1	1	−1	108.303
3	1	−1	−1	1	199.816
4	−1	1	1	−1	126.249
5	−1	1	−1	1	139.721
6	−1	−1	1	1	153.804
7	1	1	1	−1	136.639
8	1	−1	1	1	148.864
9	1	1	−1	1	176.284
10	−1	1	1	1	136.113
11	−1	−1	1	−1	144.585
12	−1	−1	−1	−1	214.265

##### 2.2.5.3 Box-Behnken design

Through PB experimental design, three main factors affecting the fermentation of lotus seeds and lily bulbs were identified: fermentation time, feed concentration, and temperature. The Box-Behnken experiment (Yazici et al., 2021) designed 17 experimental groups to analyze the polysaccharide content in the fermented solution of lotus seeds and lily bulbs. These experimental designs were created using Design-Expert 10 software by substituting the pre-maximum, maximum, and post-maximum conditions for the three factors.

### 2.3 Safety evaluation of lotus seed and lily fermented liquor

#### 2.3.1 Preparation of chicken embryo villous yolk sac membrane

In the fully automated incubator, chicken eggs are hatched for 9 days. After removing the 9-day-old eggs, perform the egg inspection and mark the air cell area. Remove unfertilized, lifeless, and deformed eggs. When preparing the villous allantoic membrane (CAM), use surgical forceps to remove the shell above the air cell, add 1 mL 0.9% NaCl solution along the edge of the air cell membrane and complete the experiment within 20 min.

#### 2.3.2 CAM Replace the eye irritation test method

The maximum tolerated concentration method was used to analyze the stimulation of lotus seed and lily fermentation broth on CAM. Each group contained 6 chicken embryos, which were exposed to negative controls (0.9% saline), positive controls (NaOH), reference controls (Texapon ASV), and the fermentation broth experimental group. A 200 μL solution prepared in advance was added to the areas of clear and uniform CAM vessels, gently rinsed after 3 min, and observed for 2 h. The blood vessels within the rubber rings of the chicken embryo membranes were examined under an electron microscope to assess any bleeding, hemolysis, or coagulation phenomena. Based on the conditions of bleeding, coagulation, and vessel dissolution, each chicken embryo was evaluated and scored according to the scoring table shown in Table 2. The irritancy and corrosiveness of the experimental groups were assessed.

**TABLE 2:** Stimuli classification table.

Grade	Spicy classification
ES ≤ 12	No/light irritation
12 < ES < 16	Moderate irritation
ES ≥ 16	Strong irritant/corrosive

#### 2.3.3 Data analysis

The experimental results were analyzed and charts were drawn using Prism GraphPad 10 software. All experimental data represent the average values from three independent experiments, expressed as mean ± standard deviation. One-way ANOVA was used for statistical analysis of the experimental results, and differences were considered statistically significant when *p* < 0.05.

### 2.4 Anti-inflammatory activity of fermented lotus seed and lily bulb liquid

RAW264.7 cells stored at −80°C were thawed and resuspended. The resulting cell suspension was inoculated into T25 culture flasks containing 25 mL complete DMEM. Cultivation was carried out under conditions of 37°C and 5% CO_2_. When the cell fusion rate reached 80–90%, the best-conditioned RAW264.7 cell suspension was aspirated and mixed with 0.4% trypan blue at a ratio of 1:1. The mixture was placed on a cell counter and observed under a microscope to prepare a HaCaT cell suspension with a density of 1 × 10^5^/mL.

#### 2.4.1 Evaluation of the safe concentration of lotus seed lily fermentation liquid to RAW264.7 cells

Prepare fermentation broth samples at concentrations of 0.625, 1.25, 2.5, 5, 10, 20, 30, and 40% (v/v) using RPMI-1640 basal medium. Add six duplicate wells to each concentration into a 96-well plate. For the control group, add only RPMI-1640 medium. After culturing for 20 h, sequentially add 50 μL MTT staining solution to each well. Discard the supernatant after 4 h, then add 150 μL of DMSO. Shake at low speed on a shaking table at room temperature for 10 min. Subsequently, measure the absorbance at 570 nm using a microplate reader, and calculate the RAW 264.7 cell viability according to Equation 1


$$C\text{e}ll\;survival\;rate\left(\%\right)=\frac{\left(A-A_{0}\right)}{\left(A_{1}-A_{0}\right)}\times 100\%$$ (1)

where letter A: absorbance value of safflower experimental group; A_0_: absorbance value of solvent blank group; A_1_: absorbance value of normal group

#### 2.4.2 RAW264.7 measurement of TNF-α (tumor necrosis factor) in cells

Dilute the standard in the TNF-α ELISA kit to concentrations of 0, 3.125, 6.25, 12.5, 25, 50, 100, and 200 pg/mL. Then, construct a TNF-α standard curve according to the kit’s instructions. Process the cell samples as directed by the kit, measure the absorbance of each sample at a wavelength of 450 nm, and determine the TNF-α content per well based on the standard curve. Set up six parallel experimental groups for each group.

#### 2.4.3 RAW264.7 determination of NO in cells

According to the instructions for the nitric oxide reagent kit, dilute the standard NaNO_2_ to concentrations of 0, 1, 2, 5, 10, 20, 40, and 60 μM, and plot a standard curve. Then process the cell samples according to the kit’s instructions, and measure the absorbance of each sample at a wavelength of 540 nm. Use the established NO standard curve to calculate the NO content (Chen et al., 2018) in each sample group.

### 2.5 Study on the antioxidant activity of lotus seed and lily fermented liquor to HACAT cell model *in vitro*

HaCaT cells stored at −80°C were thawed and resuscitated to obtain a cell suspension. Te cells were then inoculated into T25 cell culture fasks containing 3 mL of fully cultivated DMEM medium, and the cell culture was conducted at 37°C with 5% CO_2_. Upon reaching 80–90% confuency, the cells were observed, and the HaCaT cell suspension in good condition was aspirated. A 0.4% Trypan blue solution was added in a 1:1 ratio, and the mixture was dripped onto the cell counting plate. Te cell density was adjusted to 1 × 10^5^ cells/mL, and the HaCaT cell suspension was prepared accordingly. fermentation solution was diluted with basal DMEM medium at concentrations of 40, 30, 20, 10, 5, 2.5, 1.25, and 0.625%.

#### 2.5.1 HACAT establishment of oxidative injury model of cells

HACAT cells were seeded at a density of 1 × 10^5^ in six-well plates containing 2 mL DMEM of medium, with groups set as control, model, positive control, and experimental. Each well was added with 2 mL of cell suspension, and the cells were cultured for 24 h in an incubator at 37°C, 5% CO_2_, after which the supernatant was aspirated. In the control group, 2 mL DMEM of basal medium was added; the model, positive control, and experimental groups were, respectively, supplemented with 2 mL DMEM of high-glucose basal medium, 10 μg/mL Vc of DMEM high-glucose basal medium, and DMEM high-glucose basal medium containing different concentrations of lotus and lily fermentation broth, and continued to culture for 24 h. Subsequently, the supernatant was aspirated again, and the control group was supplemented with 2 mL DMEM of high-glucose basal medium, while the other groups were supplemented with 2 mL of 0.5 mmoL/L H_2_O_2_ solution, and continued to culture for 4 h.

After the observation, use 1 mL of trypsin to digest the cells, wash twice with PBS, centrifuge at 1,000 r/min for 10 min, aspirate and resuspend 1 mL PBS of the cell pellet, sonicate at 4°C for 5 min (100 W, 5 s per cycle, 20 s interval), and measure the protein concentration using the Bradford protein quantification kit. Store at 4°C for later testing, which will be used to determine the antioxidant activity of the lotus seed lily fermentation solution against HACAT oxidation.

#### 2.5.2 HACAT total antioxidant capacity (T-AOC) of cells was determined

Dilute the 10 mM Trolox solution to different concentration gradients of 0.1, 0.2, 0.4, 0.8, and 1.0 mM. Use these gradient solutions to react with the reagent IV application solution and ABTS working solution to measure the HACAT cell sample indicators. For the blank tube, add 10 μL distilled water, 20 μL reagent IV application solution, and 170 μL ABTS working solution. For the standard tube, add 10 μL of different concentration standard application solution, 20 μL reagent IV application solution, and 170 μL ABTS working solution. React at room temperature for 6 min, then measure the OD value at 405 nm. Finally, take 10 μL of cell sample and add it to a 96-well plate, sequentially adding 20 μL of reagent IV application solution and 170 μL ABTS working solution. React at room temperature for 6 min, then measure the absorbance at 405 nm. Substitute the absorbance into the standard curve to calculate the sample concentration.

#### 2.5.3 HACAT determination of catalase activity (CAT) in cells

Using a UV spectrophotometer to measure absorbance at 240 nm, calibrate with distilled water. Add 20 μL of cell sample to the cuvette, then quickly add 3 mL of substrate solution preheated to 25°C with an OD value between 0.5 and 0.55. Immediately record the absorbance at 240 nm as OD1, and after 1 min, record OD2. Substitute these two values into Equation 2 to calculate CAT activity.


Total⁢antioxidant⁢capacity= (2)

$$\frac{\mathrm{Ameauring}\,\mathrm{tube}-\mathrm{Acontrol}\,\mathrm{tube}}{0.01}÷30\times 3.7$$

#### 2.5.4 Determination of malondialdehyde (MDA) content in HACAT cells

Reagents were prepared according to the instructions of the MDA test kit. The vortex mixer was used to mix the reagent, and the reaction system was mixed with 95°C water bath for 40 min. After cooling, the reaction system was centrifuged at 4,000 r/min for 10 min, and the supernatant was taken. The absorbance value was measured at 532 nm, and the content was calculated by Equation (3).


$$\mathrm{MDA}\,\mathrm{content}=\frac{\mathrm{Measurde}\,\mathrm{OD}\,\mathrm{values}\,\mathrm{of}\,\mathrm{control}}{\mathrm{Standard}\,\mathrm{OD}\,\mathrm{values}-\mathrm{Blank}\,\mathrm{OD}\,\mathrm{value}}$$ (3)

×Concentration⁢of⁢standard

÷Protein⁢concentration⁢of⁢the⁢samples⁢tobemeasured   

#### 2.5.5 HACAT determination of superoxide dismutase (SOD) activity in cells

The preparation of the reagent is described in the instructions for the total superoxide dismutase (SOD) assay kit. The total SOD activity was calculated according to Equation (4) after the reaction was completed.


$$\text{T-SOD}\,\mathrm{viability}=\frac{\mathrm{Control}\,\mathrm{OD}-\mathrm{Measured}\,\mathrm{OD}\,\mathrm{value}}{\mathrm{Blank}\,\mathrm{OD}\,\mathrm{value}}÷50\%$$ (4)

$$\;\;\times \frac{T\,o\,t\,a\,l\,\mathrm{volume}\,\mathrm{of}\,\mathrm{reaction}\,\mathrm{solution}\,\left(\mathrm{mL}\right)}{\mathrm{Sample}\,\mathrm{size}\,\left(\mathrm{statistics}\right)\,\left(\mathrm{mL}\right)}$$

  ÷proteincontent             

### 2.6 Study on whitening activity of fermentation products on B16-F10 cell model *in vitro*

#### 2.6.1 B16-F10 cell culture

The B16-F10 cells stored at −80°C were thawed and resuspended to obtain a cell suspension. The cells were then seeded into T25 culture flasks containing 3 mL of complete DRPMI-1640 medium and cultured under conditions of 37°C and 5% CO_2_. When the cells reached 80–90% confluence, they were observed, and well-conditioned HaCaT cell suspensions were aspirated. A 0.4% trypan blue solution was added to the cell counting chamber at a 1:1 ratio. The cell density was adjusted to 1 × 10^5^ cells/mL, and the corresponding B16-F10 cell suspension was prepared. The lotus and lily fermentation broth was diluted with RPMI-1640 medium to achieve concentrations of 40, 30, 20, 10, 5, 2.5, 1.25, and 0.625%

#### 2.6.2 Determination of tyrosinase activity in B16-F10 cells

Cells were seeded at a density of 1 × 10^5^ in six-well plates containing 2 mL RPMI-1640 medium. The plates were divided into Control, Positive Control, and Experimental groups, with 2 mL of cell suspension added to each well. The plates were cultured at 37°C, 5% CO_2_ in a cell incubator for 24 h, followed by the removal of the supernatant. The Control group consisted of normal cells, the Positive Control group included 500 μg/mL kojid acid, and the Experimental group contained fermentation broth. The six-well plates were incubated at 37°C, 5% CO_2_ for 24 h. The cells were washed three times with phosphate-buffered saline (PBS), then sonicated at 4°C for 5 min (100 W, 5 s per cycle, with a 20 s interval). The supernatant was collected and centrifuged for 20 min to obtain the enzyme solution. BCA protein assay kit was used to quantify the protein content, adjusting the protein level to 20 μg. A lysis buffer containing 2.5 mM L-DOPA and 0.1 M phosphate buffer (100 μL) was transferred to the wells of a 96-well plate and incubated at 37°C for 1 h. The absorbance was measured immediately at 475 nm using a microplate reader, with each group repeated three times (Guo et al., 2020). The inhibition rate of tyrosinase activity between cells was calculated using Equation (5)


$$\begin{array}{c} \mathrm{Tyrosinase}\,\mathrm{activity}\,\mathrm{inhibition}\,\mathrm{rate}=1-\mathrm{ODa}/\mathrm{ODb}\times 100\%\\ \mathrm{ODa}:\mathrm{absorbance}\,\mathrm{of}\,\mathrm{the}\,\mathrm{experimental}\,\mathrm{group}\\ \mathrm{ODb}:\mathrm{absorbance}\,\mathrm{of}\,\mathrm{model}\,\mathrm{group}\; \end{array}$$ (5)

#### 2.6.3 Determination of the inhibition rate of melanin production in B16-F10 cells

Wash the cells with phosphate-buffered saline (PBS), and resuspend the precipitate obtained from centrifugation (12,000 × g, 10 min) in 1 mol/L NaOH of 10% DMSO in an 80°C water bath for 30 min. Measure the melanin content in each well using a microplate reader at 490 nm, with three replicates per group . Calculate the inhibitory effect on melanin production for each group according to Equation (6).


$$\begin{array}{c} \mathrm{Inhibition}\mathrm{of}\mathrm{melanin}\mathrm{production}\left(\%\right)\\ =1-\left[\left(A-\mathrm{A0}\right)/\mathrm{Ax}-\mathrm{A0}\right]\times 100\%\\ A:\mathrm{the}\,\mathrm{OD}\,\mathrm{value}\,\mathrm{of}\,\mathrm{the}\,\mathrm{experimental}\,\mathrm{group}\\ \mathrm{A0}:\mathrm{the}\,\mathrm{OD}\,\mathrm{value}\,\mathrm{of}\,\mathrm{the}\,\mathrm{blank}\,\mathrm{group}\\ \mathrm{AX}:\mathrm{the}\,\mathrm{OD}\,\mathrm{value}\,\mathrm{of}\,\mathrm{the}\,\mathrm{control}\,\mathrm{group}\; \end{array}$$ (6)

#### 2.7 Study on biological activity of lotus seed and lily fermented liquid *in vivo*

Select zebrafish embryos of the AB strain that have developed normally. Dilute the lotus and lily fermentation solution to low, medium, and high concentrations. Place 10 embryos in each well of a 24-well plate, add 1 mL of sample solution, and observe the survival rate, hatching rate, and malformation rate of the embryos at 72 hpf under a stereomicroscope. Calculate the survival rate, hatching rate, and malformation rate at 72 hpf using Equations (7–9), to select an appropriate concentration for subsequent experiments.


$$\mathrm{Mortality}\mathrm{rate}\left(\%\right)=100-\left\{\frac{\mathrm{Number}\,\mathrm{of}\,\mathrm{dead}\,\mathrm{embryos}}{\mathrm{Total}\,\mathrm{number}\,\mathrm{of}\,\mathrm{embryos}}\times 100\right\}\;$$ (7)

$$\mathrm{Hatching}\mathrm{rate}\left(\%\right)=\left\{\frac{\mathrm{Number}\,\mathrm{of}\,\mathrm{embryos}\,\mathrm{hatched}}{\mathrm{Total}\,\mathrm{number}\,\mathrm{of}\,\mathrm{embryos}}\right\}\times 100\%$$ (8)

$$\mathrm{Deformity}\mathrm{rate}\left(\%\right)=\left\{\frac{\mathrm{Number}\,\mathrm{of}\,\mathrm{deformed}\,\mathrm{embryos}}{\mathrm{Total}\,\mathrm{number}\,\mathrm{of}\,\mathrm{embryos}}\right\}\times 100\%$$ (9)

#### 2.7.1 Preparation of zebrafish assay samples

Select normally developing zebrafish embryos and place themin a 6-well plate, with 20 embryos per well. Set up the control group, positive control group, model group, and experimental group. The Control group and model group are both added 4 mL of culture medium, while the experimental group is added 4 mL of lotus and lily fermentation broth at different concentrations. The positive control group is added 4 mL of culture medium containing 10 μg/mL of Vc. After 72 hpf, except for the Control group, all other groups are added 4 mL of AAPH solution at a concentration of 2 mmol/L, and left for 6 h. Use physiological saline as the homogenizing medium to prepare tissue homogenates. Centrifuge at 4°C, 12,000 r/min for 15 min, collect the supernatant, measure protein concentration, and proceed with subsequent experiments.

#### 2.7.2 Determination of total antioxidant capacity

According to the operation guide of colorimetric method in the total antioxidant capacity kit, 10 μL samples of HaCaT cells and zebrafish were collected, 20 μL reagent four application solution and 170 μL ABTS working solution were added, and the reaction was carried out at room temperature for 6 min. The absorbance value of each well was measured at 405 nm, and the absorbance value of each sample was determined by substituting the standard curve.

#### 2.7.3 Determination of malondialdehyde content

According to the operating instructions for the TBA method in the glutaraldehyde reagent kit, mix various reagents. The reaction is carried out in a 95°C water bath for 40 min, then cooled and centrifuged at 4,500 rpm for 10 min. Collect the supernatant and measure the absorbance of each sample at a wavelength of 532 nm. Determine the glutaraldehyde content in each sample group by substituting the values into Equation (3). The content of glyoxal was measured against the standard value of blank value and the standard concentration of protein in the sample to be tested.

#### 2.7.4 Determination of superoxide dismutase activity

The determination of T-SOD activity followed the operating instructions of T-SOD kit (using WST-1 method). After the reaction was completed, the T-SOD activity of each sample group was calculated by Equation (4).

### 2.8 Study on whitening activity of lotus seed and lily fermented liquid in zebrafish model

#### 2.8.1 Determination of the relative content of melanin in zebrafish

The experiment was divided into a blank group, a positive control group (PTU), and an experimental group (with low, medium, and high concentrations of lotus and lily fermentation solutions). Zebrafish embryos were placed in a 6-well plate, with 20 embryos per well. Two milliliters of the corresponding solution were added to each well, and the culture was maintained for 48 h. The medium was changed every 12 h, and the melanin production in each group of zebrafish embryos was observed and recorded using an optical microscope. Data analysis was performed using Image J software, calculating the total area of melanin spots in different groups within the same region. The total melanin area of the Control group was set as 100%, and the relative melanin content was calculated for each treatment group. Each group was tested three times.

#### 2.8.2 Effects of tyrosinase in zebrafish

The zebrafish embryos were studied for their response to different reagents, including the Control group without reagent addition, the positive group with PTU reagent addition, and the experimental group with fermentation broth reagent addition. After a 72-h development period, the zebrafish embryos were subjected to enzyme extraction and stored at -20°C. Subsequently, the enzyme extract was reacted with 1 mmol/L L-DOPA and PBS buffer, and its absorbance was measured at 475 nm. Each experiment was repeated three times, and the tyrosinase activity inhibition rate was calculated using Equation (5).

### 2.9 Anti-inflammatory activity of lotus seed and lily fermented liquid on zebrafish model *in vivo*

#### 2.9.1 Effect of fermentation broth on inflammation caused by copper sulfate

Under the microscope, select normally developing 3 dpf Tg (corola: EGFP) transgenic zebrafish larvae and transfer them into a 6-well plate, placing 20 individuals in each well. Set up control group, model group, positive dexamethasone group, and high, medium, and low concentration fermentation solution experimental groups. The control and model groups receive no medication, while the experimental and positive groups are treated with drugs for 1 h. Afterward, except for the control group, all other groups are treated with 20 μmol/L of copper sulfate for 2 h to prepare an inflammatory model. Subsequently, observe the inflammatory cell conditions in the zebrafish under a fluorescence microscope and take photos for record, then count the number of inflammatory cells.

### 2.10 The inhibitory effect of lotus seed lily fermentation on acne pathogenic bacteria

#### 2.10.1 Preparation of solid culture medium of thioethanolate

Weigh accurately 2.90 g of thioethanolate fluid culture medium powder into a high-pressure resistant glass bottle, then add 1.50 g of agar and 100 mL of distilled water. Heat and stir until dissolved to boiling, sterilize at 121C° for 20 min in an autoclave. Inoculate on a petri dish under sterile conditions on a laminar flow hood, let it cool and solidify, then invert the plate, seal with cellophane tape, and store at 4°C in the refrigerator for later use.

#### 2.10.2 Strain activation and cryopreservation

Under sterile conditions in a laminar flow cabinet, Staphylococcus aureus and Staphylococcus epidermidis were inoculated into test tubes containing 5 mL of PDA liquid medium and cultured aerobically at 37°C for 18–24 h. Propionibacterium acnes was inoculated into test tubes containing 5 mL of liquid thioethanolate medium and placed in an anaerobic bag, where it was cultured anaerobically at 37°C for 48 h. After cultivation, the bacterial suspension was picked with an inoculating loop and streaked onto solid plates using the three-zone streaking method, and further cultured until single colonies appeared. The single colonies were then cultured to achieve a bacterial concentration of OD600 nm = 0.8∼1.2, yielding seed cultures. Using the McFarland turbidity method, the bacterial suspension was adjusted to a 0.5 McFarland turbidity (1.5 × 10^8^ cfu/mL) using liquid medium, which served as the bacterial suspension for subsequent experiments. The bacterial suspension was stored at 4°C and used within 4 h.

#### 2.10.3 Determination of minimum inhibitory concentration (MIC)

The MIC of the lotus and lily fermentation broth against the test bacteria was determined using the 2,3,5-chlorotriphenyltetrazole (TTC) method (Kahl et al., 2022). A bacterial suspension with a concentration of 0.5 McFarland turbidity was added to a 96-well plate, and blank control groups, negative control groups, positive control groups, and experimental groups were set up, respectively. In each well of the negative control group, positive control group, and experimental group, 50 μL of bacterial suspension was mixed with 50 μL of broth culture (Dai et al., 2021). The blank control group received no reagents or bacterial suspension, while the negative control group was supplemented with 100 μL of sterile 0.9% saline solution, and the positive control group was supplemented with 100 μL of 100 μg/mL minocycline (Raoof et al., 2020).

The experimental group received 100 μL of physiological saline diluted to achieve final concentrations of 100, 90, 80, 70, 60, 50, 40, 30, 20, and 10% of the lotus and lily fermentation broth. Staphylococcus aureus and Staphylococcus epidermidis were cultured in a 37°C incubator for 24 h, and Propionibacterium acnes was placed in an anaerobic bag and cultured in a 37°C incubator for 48 h. After cultivation, 20 μL of 0.2% TTC solution was added, and the color was observed after 4 h of dark cultivation to determine the MIC. Each group was tested three times.

#### 2.10.4 Determination of antibacterial activity

The K-B paper disc method was used to determine the antibacterial activity of lotus seed lily fermentation broth against three test bacteria. A bacterial suspension with a 0.5 McFarland turbidity was spread on solid media and left undisturbed for 5 min. Saturated antibiotic discs soaked in the corresponding concentration of fermentation broth were then placed on the surface of the test plates and inverted in a constant temperature incubator for cultivation. After cultivation, the size of the inhibition zone was measured using the crosshair caliper method. Three replicate experiments were conducted for each group.

## 3 Results and discussion

### 3.1 Screening of dominant strains and optimal mixing ratio

**FIGURE 1:**
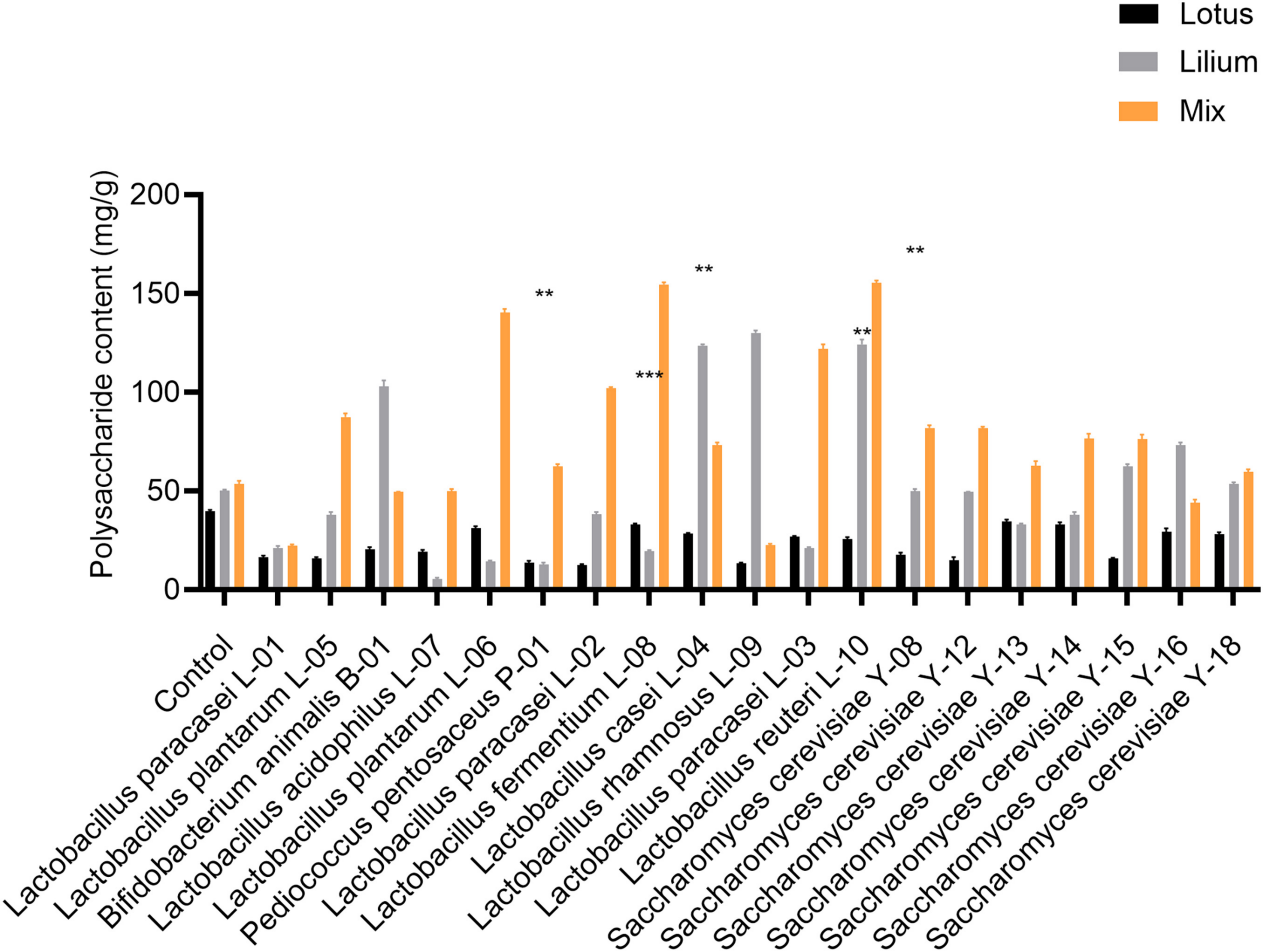
Comparison of polysaccharide content between lotus seed fermentation alone, Lilii bulbus fermentation alone and mixed fermentation of lotus seed and Lilii bulbus (compared with the Control group, **P < 0.01, ***P < 0.001).

This study first utilized microbial fermentation technology to screen fermentation methods and dominant strains, and prepared 19 different fermented lotus seed and lily bulb fermentation liquids using various probiotics. The results showed that lactic acid bacteria outperformed yeast in increasing polysaccharide content, with L-08 lactic acid bacteria showing the most significant improvement in total polysaccharide content, as shown in Figure 1. When the ratio of lily bulbs to lotus seeds was 4:2, the mixed fermentation effect was optimal.

### 3.2 Optimization of fermentation conditions

#### 3.2.1 Single factor experiment

**FIGURE 2:**
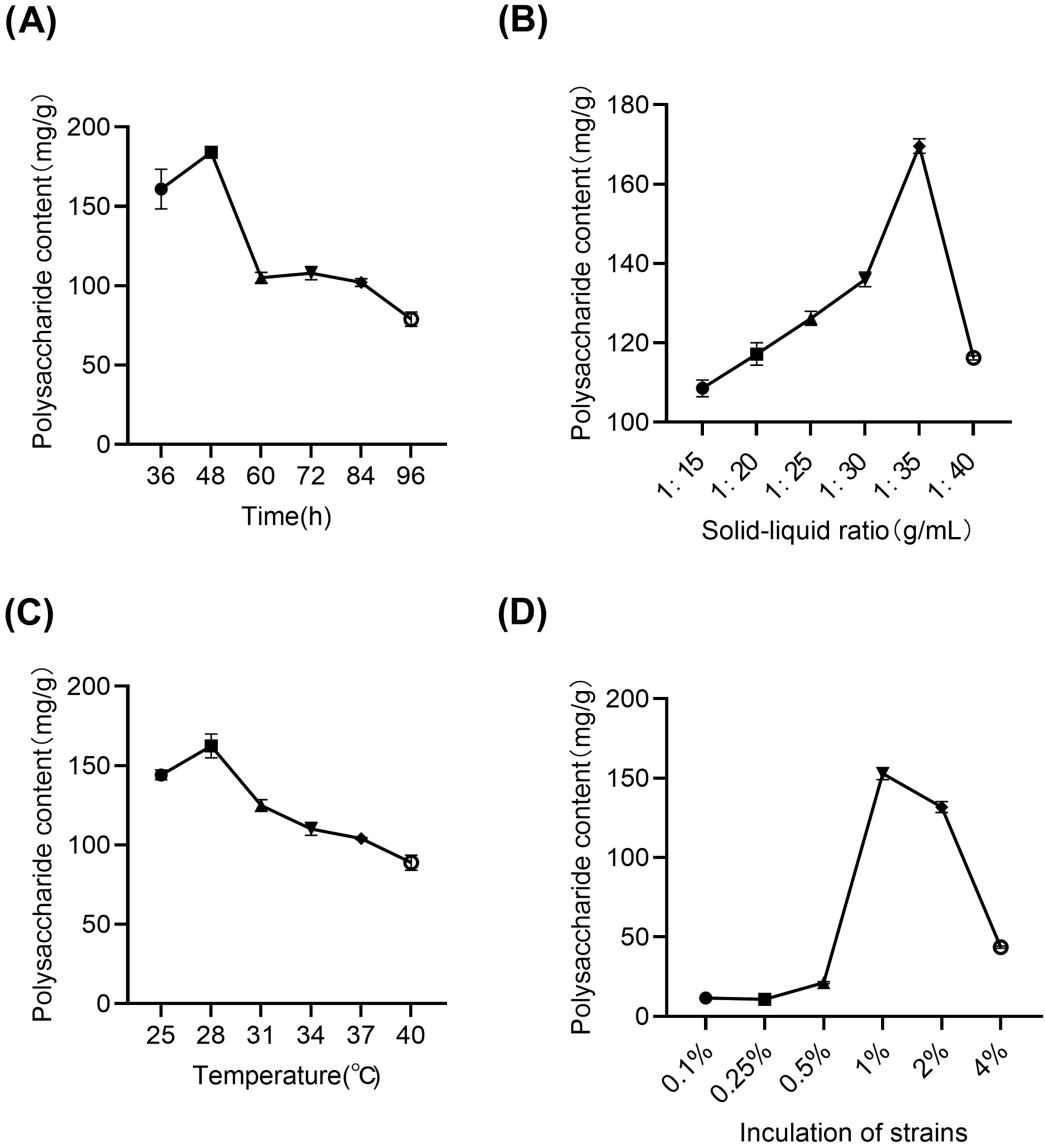
One-way experiment. **(A)** Fermentation time. **(B)** Solid–liquid ratio. **(C)** Fermentation temperature **(D)** Inoculum volume of bacterial solution. (Compared with the control group, *p < 0.05 and **p < 0.01).

The total polysaccharide content in lotus seed and lily fermented liquor was used as the index to investigate the effects of fermentation time, fermentation temperature, feed ratio and inoculation amount on the polysaccharide content in fermented liquor by single factor method. The results of single factor experiment are shown in Figure 2. Using Lactobacillus L-08 and lily bulbs to represent lotus seeds at a ratio of 4:2 as the control group, after 72 h of fermentation, the total polysaccharide content of YF-5 fermented lotus seeds and lily bulbs reached its maximum (Figure 2A). When the feed-to-batch ratio was 1:35, the total polysaccharide content in the fermented lotus bulb solution reached its highest level (Figure 2B). However, beyond this ratio, the total polysaccharide content gradually decreased. As shown in Figure 2C, when the fermentation temperature was 28°C, the total polysaccharide content in the fermented lotus bulb solution reached its peak. When the bacterial inoculum increased to 1%, the total polysaccharide content in the fermented lotus bulb solution reached its maximum. However, when the bacterial inoculum of Lactobacillus L-08 exceeded 1%, the total polysaccharide content in the fermented lotus bulb solution showed a downward trend (Figure 2D).

The effects of fermentation time, fermentation temperature, inoculation amount and feed liquid ratio on polysaccharide content were investigated by single factor method, and the optimal fermentation conditions of 1:35 (g/mL) feed liquid ratio, 1% inoculation amount and 28°C fermentation for 48 h were determined.

The research by Liu et al. (2020) proposed that substrate concentration is a key parameter in fermentation. When selecting parameters, it is necessary to balance maintaining the activity of the fermenting strain and avoiding the adverse effects of substrate overload on the quality of the fermentation product. A reasonable inoculation amount is crucial for the health and efficiency of the fermentation process; high inoculation amounts can lead to reduced polysaccharide content and growth inhibition among strains, which is consistent with the findings of Greser and Avcioglu (2022). In the mixed fermentation of lotus seeds and lily bulbs, fermentation temperature is a critical factor, as it affects the bioenzyme activity and biochemical reactions during Lactobacillus growth. This finding aligns with the results of Du et al. (2022), emphasizing the importance of regulating an appropriate fermentation temperature in the mixed fermentation of lotus seeds and lily bulbs. The metabolic activities of L-08 promote the synthesis of polysaccharides, but after a certain period, polysaccharides may begin to undergo decomposition or transformation processes. The impact of fermentation time on polysaccharide content is related to the metabolic activities of the strain and substrate utilization, which is consistent with the findings of Li et al. (2021).

#### 3.2.2 Plackett-Burman experimental results

Through the PB experiment conducted using Design-Expert 11.0 software, the polysaccharide content was measured under various experimental conditions. The system analyzed the effects of fermentation time, fermentation temperature, inoculum amount, and feed-to-liquid ratio on lotus seed and lily fermentation to determine the most significant factors affecting polysaccharide content during fermentation. Using the results from the Plackett-Burman experiment, a multiple linear regression equation R1 = +148. 91-, 18.69A-16. 37B-, 1.06C-.8.87D was established to describe the relationship between the polysaccharide content R1 in the lotus seed and lily fermentation broth and the treatment factors A, B, and C. Through regression analysis, the model demonstrated excellent fit (*R*^2^ = 0.8793) and statistical significance (*P* = 0.0025), with reliability at the 95% confidence level. In the order analysis of influencing factors, they were ranked from highest to lowest as follows: A-feed-to-liquid ratio (*P* = 0.0015), B-inoculum amount (*P* = 0.0031), D-time (*P* = 0.0475), C-temperature (*P* = 0.0814). The results are shown in Table 3.

**TABLE 3:** Analysis of variance of Plackett-Burmen test.

Factor	Sum of squares	Degrees of free	Mean square	*F-* price	*P* -price	Significant sorting
Model	8368.81	4	2092.2	12.75	0.0025	**
A-liquid ratio	4193.59	1	4193.59	25.56	0.0015	1
B-bacterial load	3217.16	1	3217.16	19.61	0.0031	2
C-temperature	13.36	1	13.36	0.0814	0.7836	4
D-time	944.69	1	944.69	5.76	0.0475	3
Residual	1148.62	7	164.09			
Sum	9517.43	11				

**Non - significance.

#### 3.2.3 Box-Behnken experimental results

**FIGURE 3:**
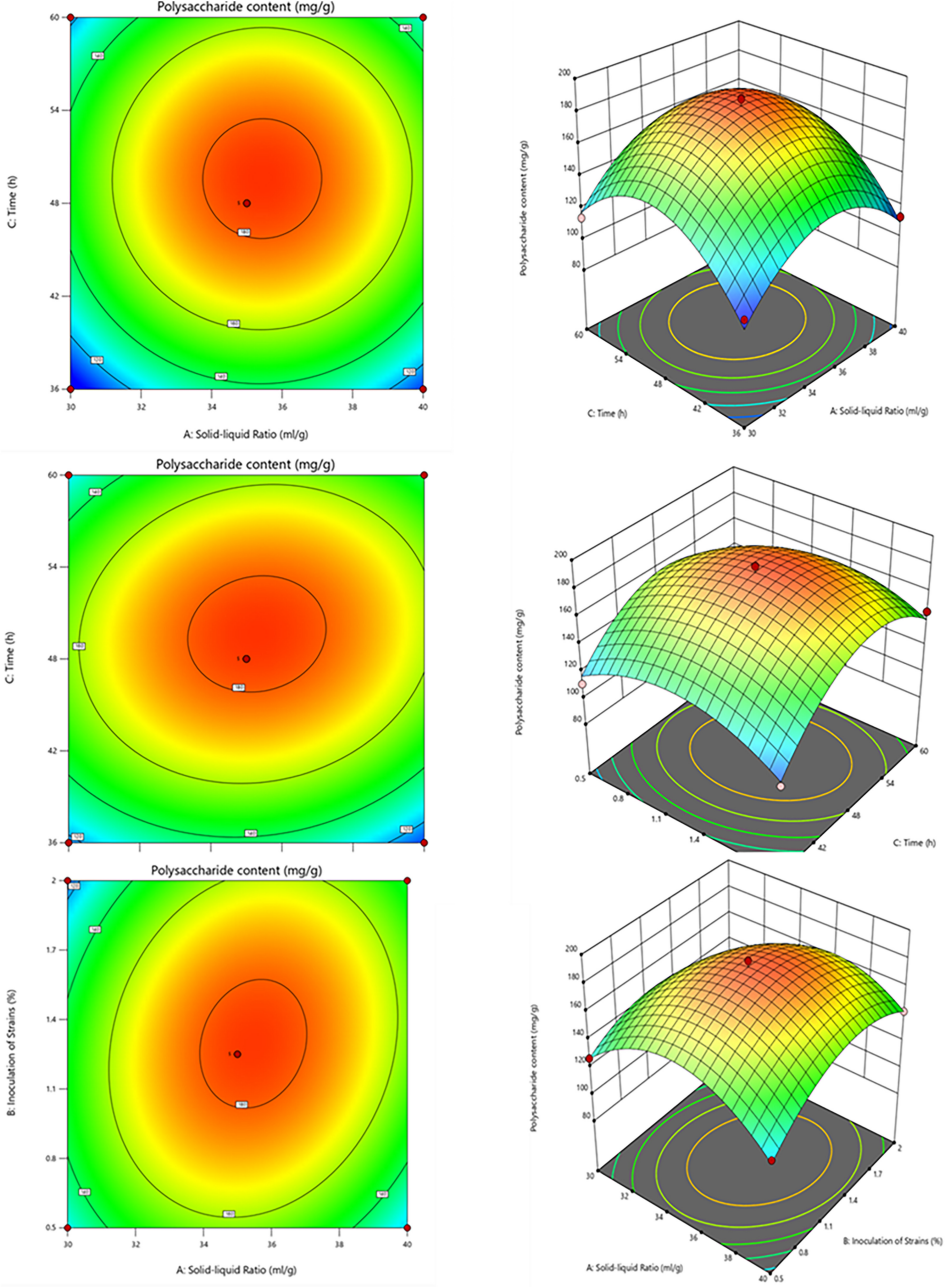
The interaction effects of solid-liquid ratio, the amount of bacteria and fermentation time on the content of polysaccharide in fermentation broth.

The Plackett-Burman (PB) experiment identified the three most significant factors affecting lotus seed lily fermentation: time, feed concentration, and temperature. Subsequently, the Box-Behnken (BB) experimental design was used to optimize these conditions. The results of Box-Behnken experimental design and results are presented in Table 4. Regression fitting was performed on the experimental data, establishing the model: R1 = +182.86+5.63A+2.09B+9.48C+8.39AB+0.6962AC+5.19BC-32.81A2-23.72B2-35.94C2, where R1 represents the polysaccharide content of the lotus seed lily fermentation broth, and A, B, and C represent the feed-to-batch ratio, inoculum amount, and fermentation time, respectively. This model shows good fit, with a coefficient of determination R2 of 0.9827 and an adjusted coefficient of determination Adj R2 of 0.9604, indicating a good match between the model and actual data. Through software analysis and prediction of the optimal levels of each factor, the result is shown in Figures 3A–C, it was found that the best optimization values are: feed-to-batch ratio 35.486 mL/g, inoculum amount 1.307%, and fermentation time 49.655 h. After averaging the results from three parallel trials, the polysaccharide content of the lotus seed lily fermentation broth was found to be 183.869 mg/g. The validation experiment results, indicate a polysaccharide content of 180.750 mg/g, which matches the predicted value, proving the successful establishment of the model.

**TABLE 4:** Box-Behnken test design and results table.

Experiment number	A	B	C	Polysaccharide content mg/g
1	0	−1	1	128.694
2	1	0	1	124.155
3	−1	−1	0	127.080
4	−1	0	1	113.885
5	0	0	0	186.945
6	1	1	0	142.355
7	0	0	0	178.889
8	1	0	−1	112.941
9	0	−1	−1	110.968
10	0	1	−1	107.336
11	−1	0	−1	105.456
12	−1	1	0	111.928
13	0	0	0	187.531
14	1	−1	0	123.935
15	0	0	0	182.631
16	0	0	0	178.303
17	0	1	1	145.808

Under optimal conditions for fermentation, the polysaccharide content increased by about 17% compared to before optimization (154.485 mg/g). This optimization process aimed to find an efficient and economical fermentation system, providing a scientific basis for the production of mixed lotus seed and lily fermentation broth. It not only facilitates large-scale preparation of bioactive components but also aligns with green production principles, offering a viable and sustainable solution for the production of functional foods and medicines. Through this research, we aim to provide more efficient and environmentally friendly fermentation processes for industrial production.

## 4 Safety assessment of lotus and lily fermentation liquid

**FIGURE 4:**
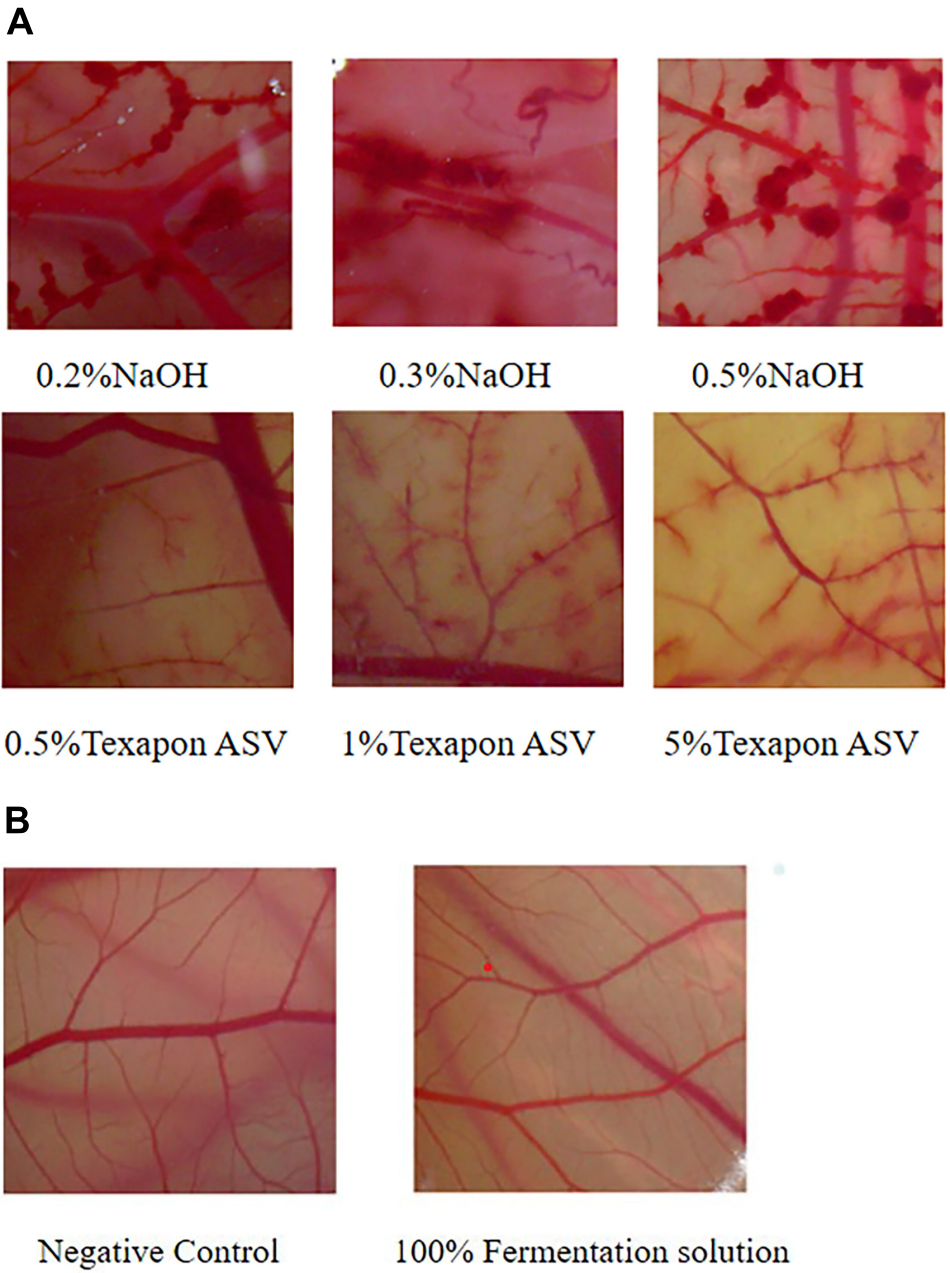
**(A)** CAM images of positive control and reference control groups. **(B)** CAM diagram of negative control and lotus seed and Lilii bulbus fermentation broth group.

This study used chicken embryo chorioallantoic membrane as an *in vitro* substitute model for eye irritation tests and evaluated the safety of lotus seed lily fermented solution using the maximum tolerated concentration method. The results showed that Texapon ASV caused varying degrees of bleeding and vasodilation at different concentrations, while NaOH led to coagulation phenomena (see Figure 4A). Next, the effects of negative control (0.9% saline) and 100% lotus seed lily fermented solution on CAM vessels were observed. The results indicated that neither the negative control nor 100% lotus seed lily fermented solution induced bleeding, coagulation, or vasodilation, and the vascular network structure and outline remained clear (see Figure 4B). Therefore, no further dilution concentration tests were required. Consequently, we concluded that the lotus seed lily fermented solution has no or mild irritant effects on the eyes, indicating it is a safe and non-irritating solution.

## 5 Study on the anti-inflammatory activity of fermented products against RAW 264.7 cell model *in vitro*

### 5.1 Effects of lotus seed and lily fermentation on cell activity of RAW 264.7

**FIGURE 5:**
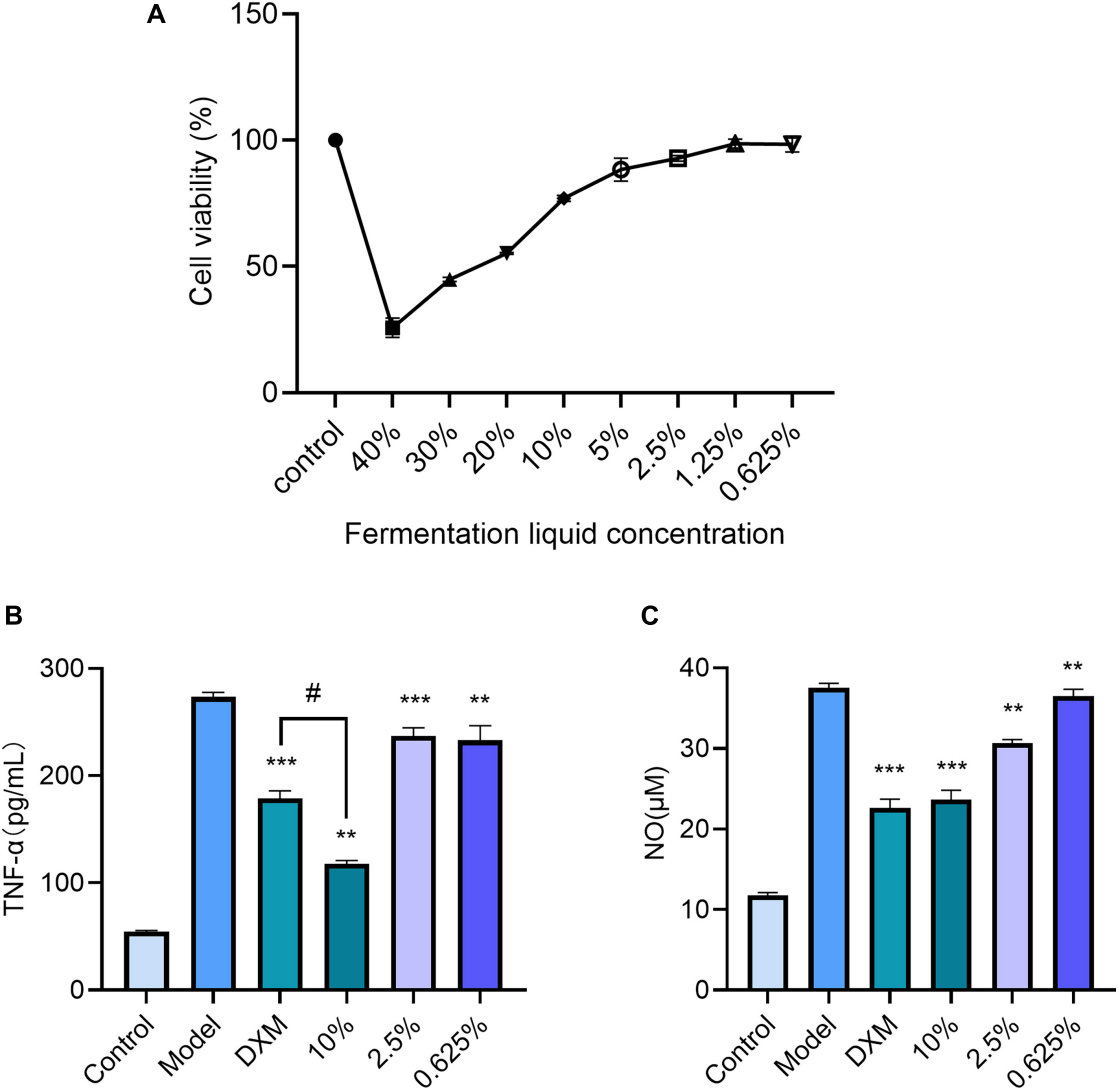
**(A)** Effects of different concentrations of lotus seed Lilii bulbus fermentation broth on the survival rate of RAW246.7 cells. **(B)** TNF-α content (Compared with the model group, **P < 0.01, ***P < 0.001, #*P <* 0.05). **(C)** NO content (Compared with the model group, **P < 0.01, ***P < 0.001).

The relative cell viability of RAW 264.7 cells was determined using the MTT method (Buranaamnuay, 2021) under different treatment groups, as shown in Figure 5A. The cell viability of the Control group was set at 100%, and the lotus and lily fermentation broth at 20–40% concentration had a significant impact on RAW 264.7 cells, with relative cell viability below 70%. In contrast, the lotus and lily fermentation broth at 0.625–10% concentration had a lesser impact on RAW 264.7 cells, with relative cell viability above 70%. Therefore, the concentration of the lotus and lily fermentation broth used as a cell inflammation model with RAW 264.7 cells is 0.625–10%.

### 5.2 Effect of lotus seed and lily fermentation on TNF-α (tumor necrosis factor) content in RAW 264.7 cells

Matured LPS-stimulated mouse macrophages RAW 264.7 were used as an *in vitro* cell inflammation model to measure the changes in TNF-α levels after treatment with different concentrations of lotus and lily fermented broth. The standard curve obtained was y = 0.0088 x + 0.0767 (*R*^2^ = 0.9979), and the established TNF-α standard curve is reliable within the 95% CI. Compared to the Control group (54.456 pg/mL), the model group (273.301 pg/mL) showed a TNF-α level increase of approximately 5.02 times, indicating successful construction of the cell inflammation model. Compared to the model group, when the lotus and lily fermented broth concentration was 10% (117.752 pg/mL), the TNF-α level significantly decreased, showing a significant difference (*P* < 0.001). These results indicate that as the concentration of lotus and lily fermented broth increases, the TNF-α level in the cell inflammation model gradually decreases. At a concentration of 10%, the reduction effect is more pronounced compared to the positive group (178.888 pg/mL), as shown in Figure 5B, which has a statistically significant difference *(P* < 0.05). This suggests that the lotus and lily fermented broth has an inhibitory effect on inflammation, and the effect is significant.

### 5.3 Effect of lotus seed and lily fermentation solution on NO content in RAW 264.7 cells

By measuring the NO content in cell inflammation models treated with lotus and lily fermentation solutions of different concentrations, a standard curve was obtained: y = 0.0038 x + 0.0811 (*R*^2^ = 0.9906). The established NO standard curve is reliable within the 95% CI. Plugging the absorbance values of NO from each experimental group into the standard curve yields results as shown in Figure 5C, indicating that the lotus and lily fermentation solution can reduce the NO content in cells, particularly at a concentration of 10%, where the effect is significant and shows a highly significant difference (*P* < 0.001).

## 6 Study on the antioxidant activity of fermented products to HACAT cell model *in vitro*

### 6.1 Effect of lotus seed and lily fermented liquid on HACAT cell activity

**FIGURE 6:**
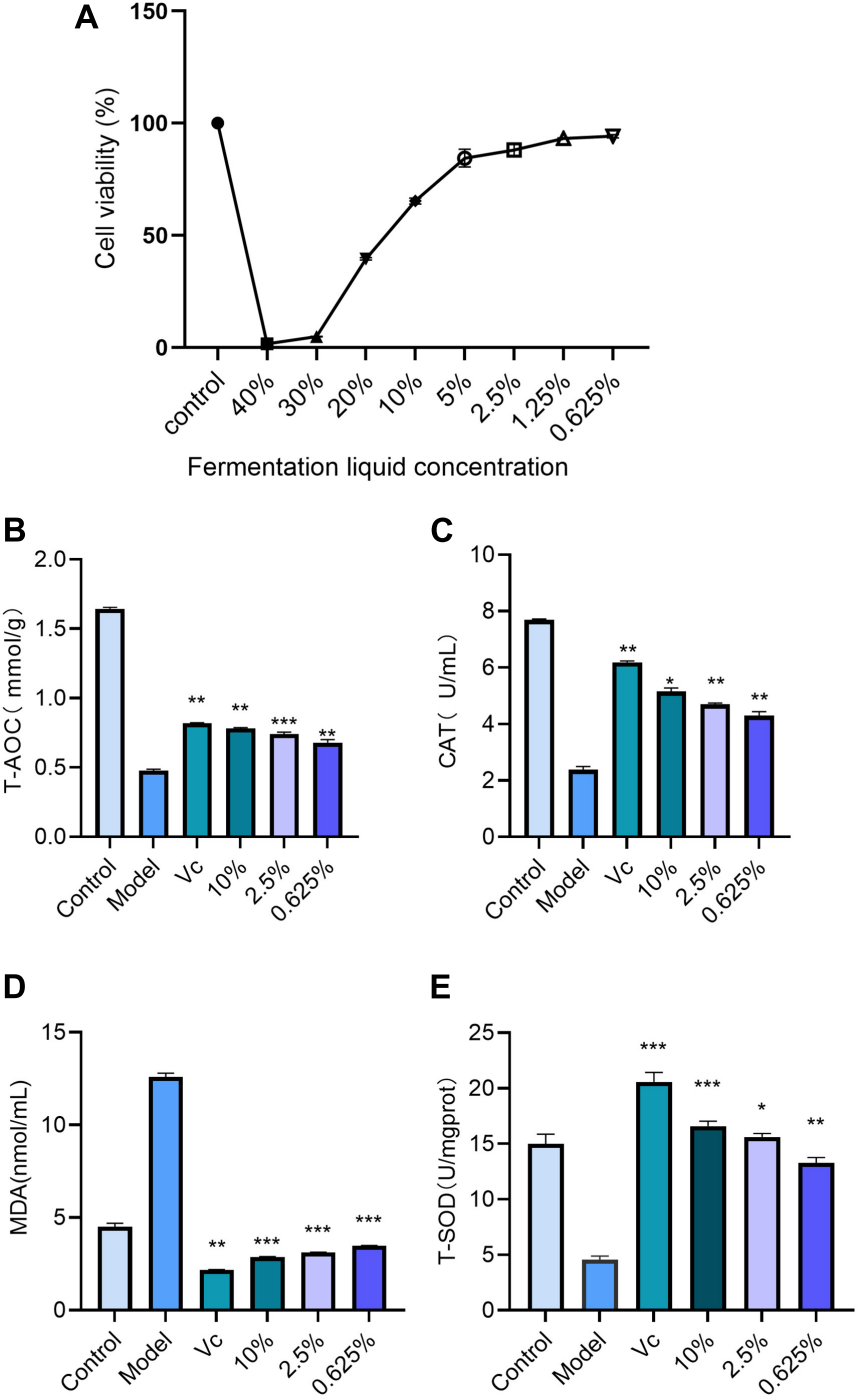
**(A)** HACAT relative cell viability. **(B)** Total antioxidant capacity of HACAT cells Compared with the model group, **P < 0.01, ***P < 0.001). **(C)** CAT activity in HACAT cells (Compared with the model group, *P < 0.05, **P < 0.01). **(D)** MDA content in cells (Compared with the model group, **P < 0.01, ***P < 0.001). **(E)** SOD activity in cells (Compared with the model group, *P < 0.05, **P < 0.01, ***P < 0.001).

The MTT method was used to determine the viability of HACAT cells at different concentrations of lotus and lily fermentation broth, as shown in Figure 6A. The relative cell viability of Group Control HACAT cells was set as 100%, while the relative cell viability of the 0.625–10% fermentation broth group was above 70%. Compared to Group Control, the relative cell viability of the 20–40% fermentation broth group was below 70%. Therefore, the concentration of the lotus and lily fermentation broth for conducting antioxidant experiments using HACAT cells is set at 0.625–10%.

### 6.2 Effects of lotus seed and lily fermentation on total antioxidant capacity, catalase activity, malondialdehyde content and superoxide dismutase activity of HaCaT cells

The total antioxidant capacity of the HACAT cell model established by measuring H_2_O_2_-induced oxidative damage was determined under different treatments. The standard curve is y = −1.0447 × + 1.511 (*R*^2^ = 0.9951), which is reliable within the 95% CI. By applying each group to the standard curve, the total antioxidant capacity of HACAT cells under different treatments was obtained. As shown in Figure 6B, compared with the Control group, the total antioxidant capacity of the model group decreased by about 70%, indicating that the HACAT cell model was successfully established. This suggests that the fermented lotus seed and lily water may enhance the cellular antioxidant defense system, thereby providing better resistance to oxidative damage.

The activity of catalase (CAT) was measured in the oxidative damage model of cells from different groups. The experimental results (Figure 6C) show that compared to the Control group, the CAT enzyme activity in the model group decreased by approximately 3.5 times, indicating the successful establishment of an HACAT cell oxidation model. As the concentration of lotus seed lily fermentation solution increased, the CAT enzyme activity gradually increased, suggesting that the lotus seed lily fermentation solution has significant antioxidant properties.

Through different cell oxidation injury models, the content of malondialdehyde (MDA) was measured. The experimental results showed that compared to Group Control, the MDA content in the model group increased by nearly 2.72 times (Figure 6D), confirming the successful establishment of the HACAT cell oxidation model. As the concentration of lotus seed lily fermentation solution increased, the MDA content in HACAT cells gradually decreased. Compared to the model group, the lotus seed lily fermentation solution group showed significant differences in MDA content (*P* < 0.01, *P* < 0.001), verifying the effectiveness of lotus seed lily fermentation solution as a potential antioxidant.

The activity of SOD was measured in different cell oxidative injury models, with the results shown in Figure 6E. Compared to the Control group, the SOD enzyme activity in the model group decreased by nearly three times, confirming the successful construction of the HACAT cell oxidative model. As the concentration of lotus seed lily fermentation broth increased, the SOD enzyme activity gradually rose. Compared to the model group, the lotus seed lily fermentation broth group showed significant statistical differences in SOD enzyme activity, suggesting that the lotus seed lily fermentation broth may enhance the biological defense against oxidative damage through positive regulation of SOD enzyme activity.

### 6.3 Study on whitening activity of fermentation products on B16-F10 cell model *in vitro*

#### 6.3.1 Effect of lotus seed lily fermentation on B16-F10 cell activity

**FIGURE 7:**
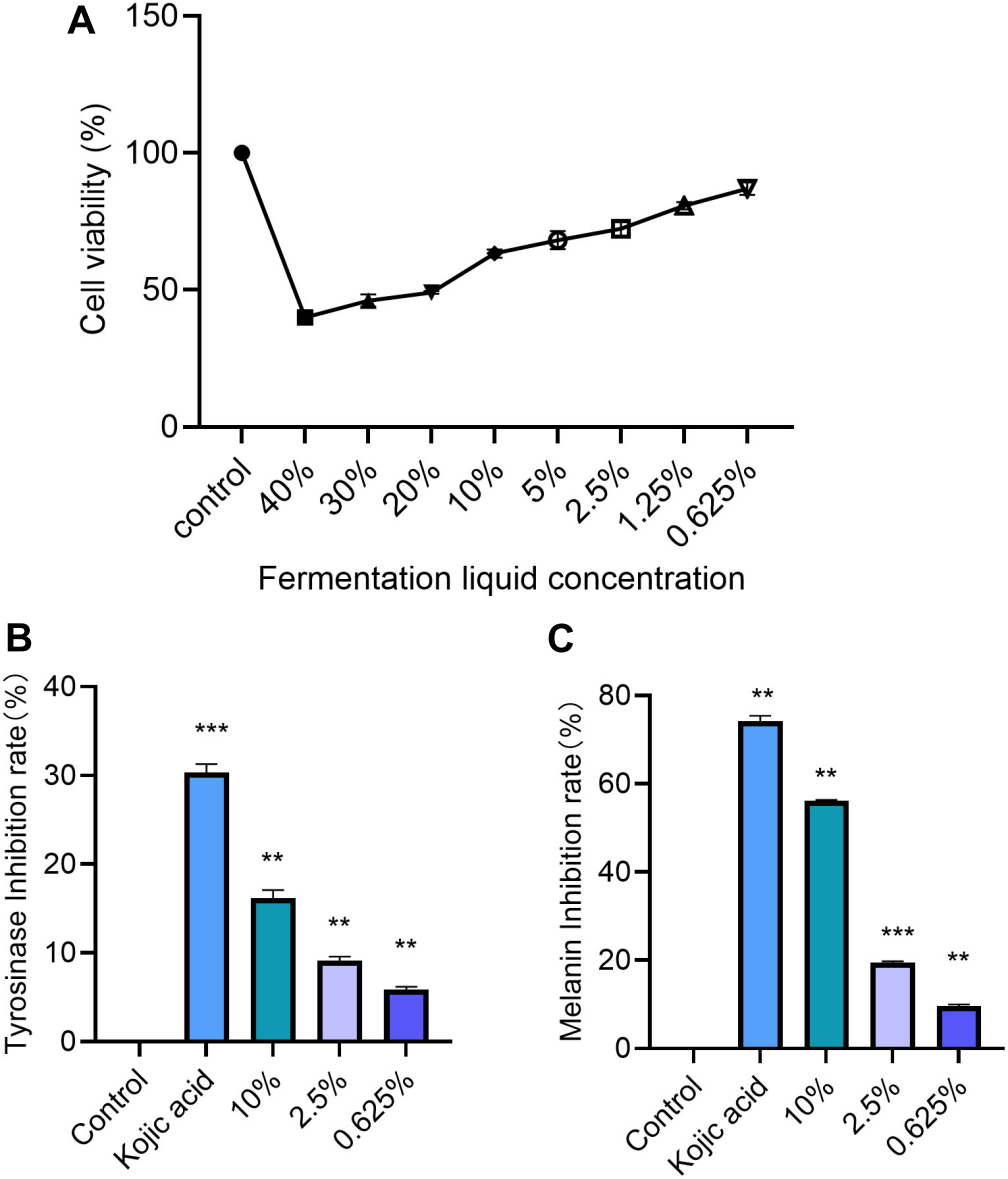
**(A)** Effects of different concentration of ginseng fermentation broth on survival rate of B16-F10. **(B)** Tyrosinase inhibition in cells (comparison with control group, **P < 0.01, ***P < 0.001). **(C)** Melanin inhibition rate in cells (comparison with model group, **P < 0.01, ***P < 0.001).

Through the MTT method, we screened the appropriate concentration range of lotus seed lily fermentation broth on B16-F10 melanoma cells (Han et al., 2020). The results showed that, relative to the cell viability of the Control group (100%), as the concentration of lotus seed lily fermentation broth increased, the cell viability of B16-F10 cells gradually decreased. When the concentration of lotus seed lily fermentation broth was between 0.625 and 10%, the relative cell viability did not significantly decrease, remaining above 60%; however, when the concentration exceeded 10%, the cell viability significantly declined. Therefore, we determined the concentration range of 0.625–10% (Figure 7A), within which the lotus seed lily fermentation broth not only did not significantly affect cell viability but also showed a trend of inhibiting the proliferation of B16-F10 melanoma cells.

#### 6.3.2 Effect of lotus seed lily fermentation on tyrosinase activity in B16-F10 cells

The experimental results show that the lotus and lily fermented solution, with gradually increasing concentration, significantly enhances the inhibition rate of tyrosinase in B16-F10 cells, as shown in Figure 7B. Compared to the control group, all groups with different concentrations of the lotus and lily fermented solution exhibited significant differences (*P* < 0.01). The positive control group, which was treated with curcumin, also significantly increased the inhibition rate of tyrosinase (*P* < 0.001). This finding fully confirms the inhibitory effect of the lotus and lily fermented solution on tyrosinase, thus verifying its potential whitening effects.

#### 6.3.3 Effect of lotus seed lily fermentation solution on the inhibition rate of melanin growth in B16-F10 cells

We evaluated the impact of lotus seed lily fermentation broth on melanin production by measuring the melanin inhibition rate of B16-F10 melanocytes . The experimental results showed that as the concentration of the lotus seed lily fermentation broth increased, the melanin inhibition rate gradually rose. All experimental groups exhibited a decreasing trend in melanin content within B16-F10 cells compared to the control group (Figure 7C).

## 7 Study on biological activity of lotus seed and lily fermented liquid

### 7.1 Determination of safe concentration of lotus seed lily fermentation solution

**FIGURE 8:**
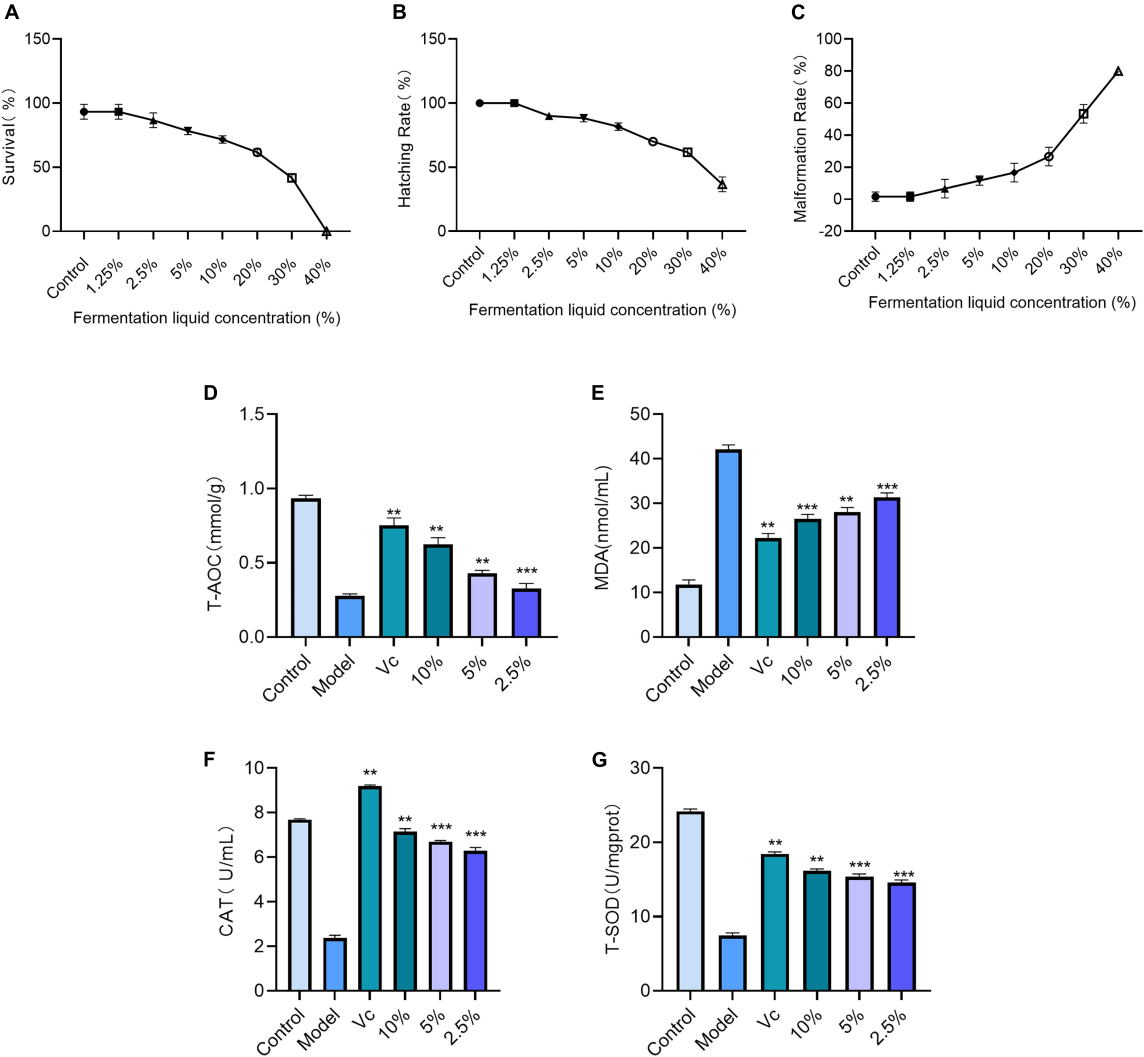
**(A)** Effects of fermentation broth on embryonic survival rate of zebrafish. **(B)** Effects of fermentation broth on the incubation rate of zebrafish embryos. **(C)** Effects of fermentation broth on the rate of embryonic deformities of zebrafish. **(D)** Total antioxidant capacity of vine tea fermentation broth (Zebrafish). (Comparison with model group, **P < 0.01, ***P < 0.001). **(E)** Influence of fermentation broth on MDA content in Zebrafish (comparison with model group, **P < 0.01, ***P < 0.001). **(F)** CAT activity in zebrafish (compared with model group, **P < 0.01, *** < 0.001). **(G)** Effect of fermentation broth on SOD activity in Zebrafish (comparison with model group, **P < 0.01, ***P < 0.001).

According to Figure 8A, the survival rate of zebrafish embryos in the lotus lily fermentation broth at concentrations ranging from 1.25 to 40% gradually decreases, with a survival rate of zero at 40%, and over 50% at concentrations below 20%. In Figure 8B, the hatching rates of zebrafish embryos under different concentrations of lotus lily fermentation broth show that the hatching rate exceeds 80% at concentrations below 10%. Figure 8C also illustrates the malformation rates of zebrafish embryos under different concentrations of lotus lily fermentation broth, showing that compared to Group Control, the malformation rate is below 20% at concentrations below 10%. Comprehensive analysis indicates that most zebrafish can achieve normal development within the concentration range of 1.25–10%. Therefore, it can be determined that the suitable concentration range for using lotus lily fermentation broth in antioxidant experiments with zebrafish embryo models is 1.25–10%.

### 7.2 Effects of lotus seed and lily fermentation on total antioxidant capacity in zebrafish

By substituting the data from each group into the T-AOC standard curve plotted in 5.4.2.2, we obtained quantified results for zebrafish total antioxidant capacity under different processing conditions, as shown in Figure 8D. Compared to the model group, all fermentation broth groups exhibited significant statistical differences in T-AOC (*P* < 0.01, *P* < 0.001). The T-AOC content showed a clear dose-dependent effect across different experimental groups, with the 10% fermentation broth concentration group showing significantly higher T-AOC levels compared to other groups.

#### 7.2.1 Effects of lotus seed and lily fermentation on MDA content in zebrafish

In the AAPH-induced zebrafish oxidative injury model (Abbate et al., 2021), we measured the changes in malondialdehyde (MDA) levels in zebrafish treated with lotus and lily fermented broth to evaluate its *in vivo* antioxidant activity. Figure 8E shows the corresponding results. As the concentration of lotus and lily fermented broth increased, the MDA levels in zebrafish gradually decreased, showing significant differences compared to the model group (*P* < 0.01, *P* < 0.001). This indicates that lotus and lily fermented broth has an antioxidant protective effect on the AAPH-induced zebrafish oxidative injury model.

#### 7.2.2 Effects of lotus seed and lily fermentation on supermutant peroxidase CAT activity in zebrafish

In the zebrafish oxidative injury model induced by AAPH, we measured the changes in superoxide dismutase (CAT) activity in zebrafish after treatment with lotus and lily fermentation broth, as shown in Figure 8F. The experimental groups exhibited a certain degree of concentration dependence, with CAT activity gradually increasing as the concentration of lotus and lily fermentation broth increased. Compared to the model group, all experimental groups showed significant differences (*P* < 0.01, *P* < 0.001), highlighting the significant effect of lotus and lily fermentation broth in enhancing zebrafish resistance to free radicals.

#### 7.2.3 Effects of lotus seed and lily fermentation on SOD activity in zebrafish

In the AAPH-induced zebrafish oxidative injury model, we measured the changes in superoxide dismutase (SOD) activity in zebrafish after treatment with lotus and lily fermentation broth, as shown in Figure 8G. Significant differences were observed compared to the model group in all experimental groups (*P* < 0.01, *P* < 0.001), indicating that the lotus and lily fermentation broth significantly enhanced the zebrafish’s resistance to free radicals.

### 7.3 Study on whitening activity of lotus seed and lily fermentation liquid on zebrafish model *in vivo*

#### 7.3.1 Determination of relative melanin content in zebrafish

**FIGURE 9:**
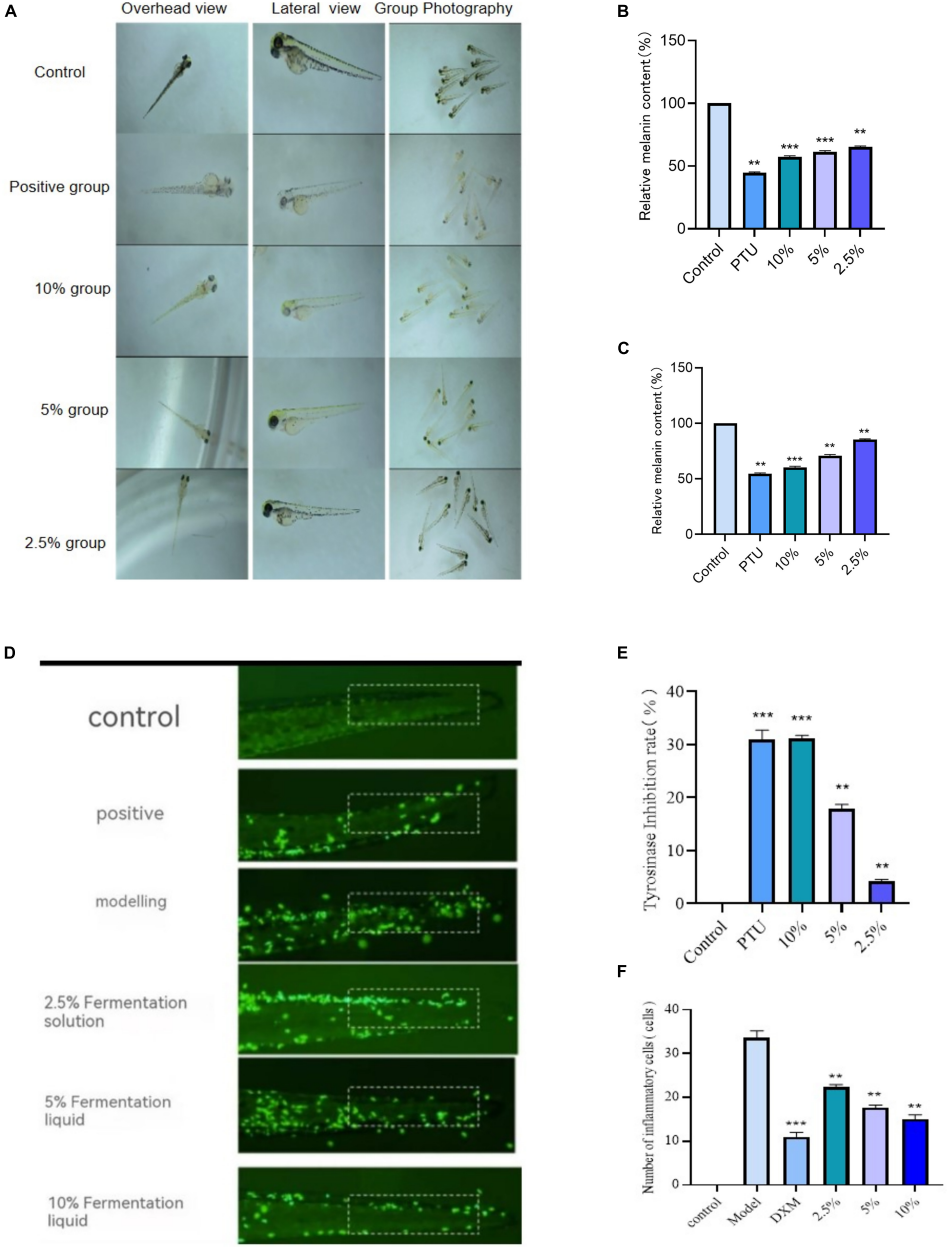
**(A)** Effects of lotus and lily fermentation on melanin production in zebrafish, melanin deposition in zebrafish larvae. **(B)** Relative melanin content on the side;relative melanin content on the back (compared with group Control, **P < 0.01, ***P < 0.001). **(C)** Effect of vine tea fermentation broth on tyrosinase inhibition rate in zebrafish (comparison with model group, **P < 0.01, ***P < 0.001). **(D)** Effects of lotus seed and Lilii bulbus fermentation broth on inflammatory factors in zebrafish. **(E)** the inflammatory site map of zebrafish larvae under fluorescence microscope. **(F)** The number of inflammatory cells in the inflammatory site (comparison with model group, **P < 0.01, ***P < 0.001).

By using Image J software for statistical analysis of imaging results, we evaluated the melanin production in zebrafish exposed to different concentrations of lotus and lily fermentation broth and positive controls. The results are shown in Figure 9. Figure 9A illustrates the deposition of melanin in different body parts. As the concentration of the lotus and lily fermentation broth gradually increased, the melanin production showed a decreasing trend, with melanin deposition gradually decreasing. This indicates that the lotus and lily fermentation broth has an inhibitory effect on melanin synthesis in zebrafish.

Figure 9B shows the comparison of melanin relative content on the side of zebrafish larvae. As the concentration of lotus and lily fermentation broth increases, there is also a trend of gradually inhibiting melanin production. Compared with Group Control, the fermentation broths in all groups significantly reduced the melanin content in zebrafish (*P* < 0.01, *P* < 0.001).

Figure 9C shows the trend of melanin relative content in the back of zebrafish larvae, which is consistent with the side melanin relative content. These experimental results consistently show that lotus seed lily fermentation liquid can significantly inhibit the synthesis of melanin in zebrafish larvae and has obvious whitening activity.

#### 7.3.2 Effects of lotus seed and lily fermentation on tyrosinase activity in zebrafish

The results shown in Figure 9D indicate that as the concentration of lotus and lily fermentation broth increases, the inhibition rate of tyrosinase gradually improves. Compared to the model group, both the lotus and lily fermentation broth group and the positive group significantly increased the inhibition rate of tyrosinase (*P* < 0.01, *P* < 0.001). This suggests that in zebrafish, the lotus and lily fermentation broth has a significant inhibitory effect on tyrosinase activity.

### 7.4 Anti-inflammatory activity of lotus seed and lily fermentation liquid on zebrafish model *in vivo*

#### 7.4.1 Inflammatory effects of lotus seed lily fermentation on zebrafish model *in vivo*

The zebrafish inflammation model induced by the copper sulfate method (Singh et al., 2022) was used to evaluate the anti-inflammatory effects of lotus seed lily fermentation broth, as shown in Figures 9E,F. Under fluorescence microscopy, significant inflammatory cell aggregation and extravasation trends were observed in all regions of the model group. However, the lotus seed lily fermentation broth and the positive control significantly reduced the migration of inflammatory cells, with a decrease in the mean cell count at the tail, showing a significant difference compared to the model group *(P* < 0.01, *P* < 0.001). This indicates that the lotus seed lily fermentation broth has demonstrated significant anti-inflammatory activity in inhibiting zebrafish inflammation.

## 8 Inhibition of acne-causing bacteria by lotus seed and lily fermentation liquid

### 8.1 Minimum inhibitory concentration (MIC) of lotus seed and lily fermentation solution

The TTC was used as an indicator (Tanaka et al., 2021) to determine the minimum inhibitory concentration (MIC) of lotus seed lily fermentation solution on the test bacteria, and the results were listed in Table 5. For *Staphylococcus epidermidis* and *Staphylococcus aureus*, the MIC of lotus seed lily fermentation solution was 30%; for Propionibacterium acnes, the MIC was 40%.

**TABLE 5:** Determination of minimum inhibitory concentration of three acne pathogens in lotus seed and Lilii bulbus fermentation broth by TTC method.

	Negative	Positive	100%	90%	80%	70%	60%	50%	40%	30%	20%	10%
Staphylococcus epidermidis	+	−	−	−	−	−	−	−	−	−	+	+
Propionibacterium acnes	+	−	−	−	−	−	−	−	−	+	+	+
SA	+	−	−	−	−	−	−	−	−	−	+	+

**FIGURE 10:**
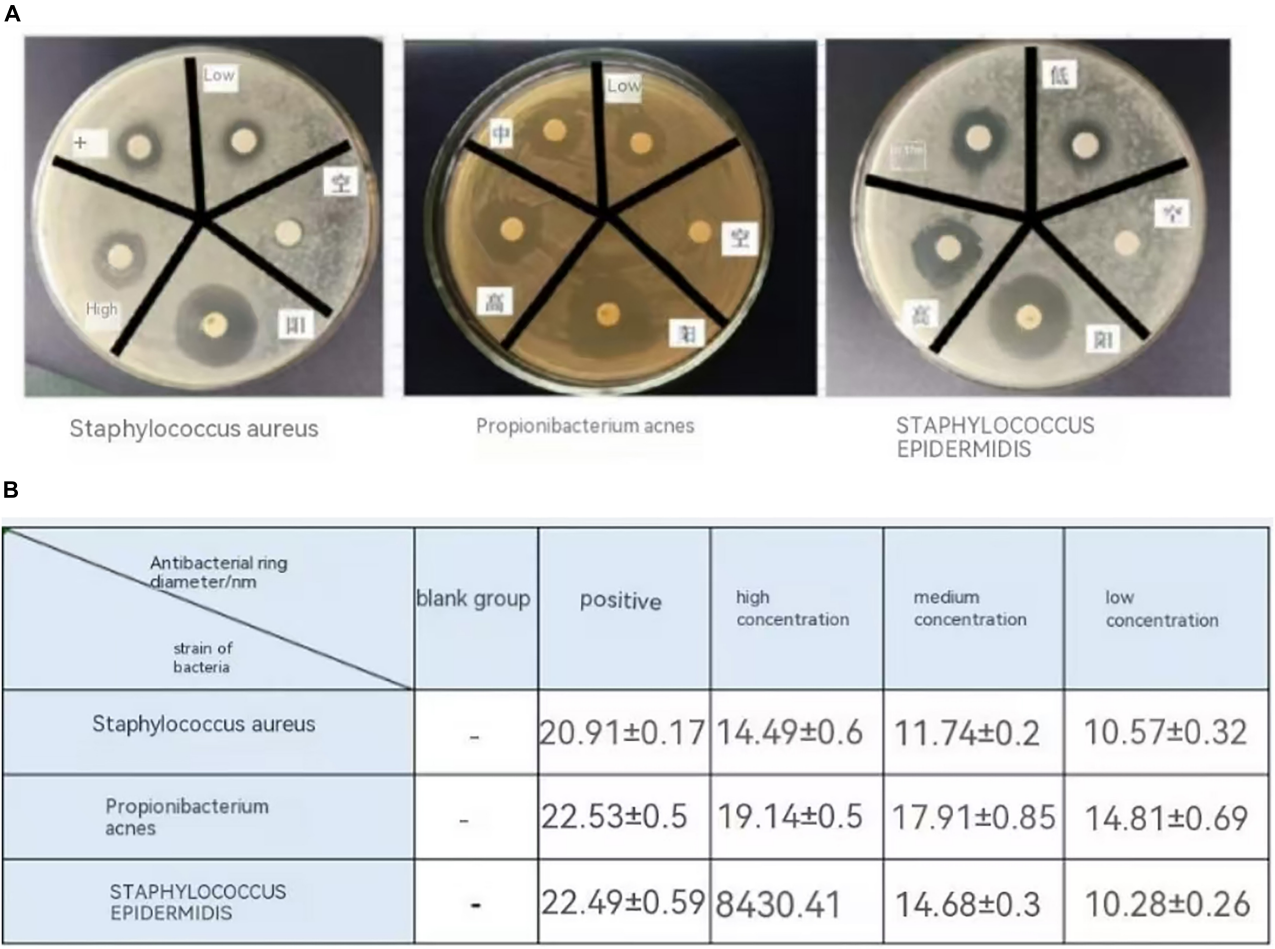
**(A)** Determination of the inhibitory activity of lotus seed and Lilii bulbus fermentation broth against three acne pathogens by K-B method. **(B)** The inhibition zone size of lotus seed and Lilii bulbus fermentation broth against three acne pathogens.

In the K-B paper disc method experiment, we set different concentrations for antibacterial effects. For *Staphylococcus epidermidis and Staphylococcus aureus*, the low, medium, and high concentrations were set at 30, 50, and 70%, respectively; for Propionibacterium acnes, the corresponding concentrations were set at 40, 60, and 80%. The MIC data in Figure 10A represent an initial assessment of antibacterial efficacy.

### 8.2 Antibacterial activity of lotus seed and lily fermented liquor

Figure 10 illustrates the inhibitory activity of lotus and lily fermented solutions at different concentrations on three common *acne-causing bacteria* using the K-B paper disc method. The specific inhibition zone sizes are shown in Figure 10B. The inhibition zones for different strains in the low, medium, and high concentration groups, as well as the positive and negative control groups, reflect the inhibitory effects of the lotus and lily fermented solution on these three pathogens. Particularly at high concentrations, the inhibition zone for Propionibacterium acnes was (19.14 ± 0.58) mm, demonstrating the most significant effect. These data strongly support the potential application of lotus and lily fermented solutions in inhibiting acne-causing bacteria.

## 9 Conclusion

Most of the current research on herbal fermentation mainly focuses on single herbal raw materials, or only conducts simple compound fermentation of conventional herbs, and mostly concentrates on the fields of food and health products, with insufficient exploration of component transformation and medical aesthetic applications. This study has for the first time achieved the scientific compound and synergistic fermentation of lotus seeds and lilies, breaking through the shortcomings of single raw material fermentation activity and poor synergistic components in conventional compound fermentation, and filling the research gap in this field. In metabolite levels, different from traditional research only focus on a single herbs total polyphenols, flavonoids, polysaccharides and other categories of component content, this study through mixed fermentation, the specificity of the induced single raw materials fermentation cannot synthesize functional metabolites, including highly active phenolic acid derivatives, anti-inflammatory oligopeptide, repair type segments of polysaccharide, etc., has been clear about the body composition and conversion mechanism of uniqueness, It provides the core material basis for the exertion of efficacy. In terms of application fields, this study focuses for the first time on the application value of this fermentation product in the fields of cosmetics and medical aesthetics. Based on its unique metabolites, it clarifies its exclusive medical aesthetic effects such as anti-skin photoaging, repairing the damaged barrier after medical aesthetics, soothing sensitive skin, fading pigmentation, and repairing the skin barrier, breaking through the limitation of the single application scenarios of traditional herbal fermentation products. It provides a brand-new direction for the development of medicinal and edible herbal fermented products in the field of medical aesthetics, featuring both academic and application innovation.

This study first applied microbial fermentation technology to optimize fermentation protocols and screen dominant strains, and 19 distinct fermented products of lotus seed and lily were prepared using different probiotics. The results demonstrated that different probiotics posed distinct effects on the accumulation of functional components in lotus seed and lily fermentation systems. Compared with yeast, lactic acid bacteria displayed superior performance in enhancing polysaccharide content, and lactic acid bacteria L-08 exhibited the most remarkable promoting effect on the water-soluble total polysaccharides derived from lotus seed and lily. The highest accumulation of water-soluble total polysaccharides was achieved under the mixed fermentation condition with a lily-to-lotus seed ratio of 4:2, which provides a reliable basis and technical support for the in-depth development and utilization of fermented lotus seed and lily products.

Secondly, we optimized the process parameters for preparing lotus seed and lily fermentation broth. Through single-factor analysis and response surface methodology, we determined the optimal fermentation conditions. The results showed that the best optimization values for each factor were a feed-to-batch ratio of 35.486 mL/g, a bacterial addition rate of 1.307%, and a fermentation time of 49.655 h. Under these conditions, the total polysaccharide content in the lotus seed and lily fermentation broth was the highest, offering advantages such as being green, low-consumption, and easy to operate, making it suitable for industrial application.

Subsequently, we evaluated the safety of the fermentation products under optimized conditions in the CAM model. The results showed that the fermented liquid of lotus seed and lily was non-stimulating to CAM at 100% concentration, indicating that the fermentation broth exhibited relatively good safety toward CAM, and no obvious toxicity was observed.

In cell experiments, we determined the safe concentration range of lotus seed lily fermentation liquid for various cells to be 0.625∼10%, and further evaluated its multiple biological activities. In the process of discussing the functional activity of fermented lotus seed and lily products, the experimental results of *in vitro* cell models and *in vivo* animal models provide crucial supporting evidence for the biological activity of the fermented products. *In vitro*, the human immortalized keratinocyte (HaCaT) oxidative damage model was applied to evaluate the antioxidant activity of the fermented products, and the results verified that the fermented lotus seed and lily products could effectively alleviate oxidative stress injury in HaCaT cells, reduce intracellular reactive oxygen species levels, and enhance the activity of antioxidant enzymes, which further confirmed the antioxidant function of the water-soluble total polysaccharides as the core active component in the fermented products. Meanwhile, zebrafish larvae and embryo models were introduced for *in vivo* functional verification. The developmental status of zebrafish embryos, the survival rate and morphological indicators of zebrafish larvae, as well as the *in vivo* antioxidant and oxidative stress repair-related indicators were systematically detected. The results showed that the fermented lotus seed and lily products could promote the normal development of zebrafish embryos, improve the survival rate of zebrafish larvae under oxidative stress, and exert significant antioxidant and oxidative damage protective effects *in vivo*. In the HACAT cell oxidation model, the lotus seed lily fermentation liquid significantly enhanced total antioxidant capacity; in the RAW 264.7 macrophage cell model, it successfully inhibited the production of inflammatory mediators, particularly in the TNF-α anti-inflammatory experiment, where the 10% concentration group showed better results than the positive drug (DXM group); in the B16-F10 melanoma cell model, it inhibited the melanin synthesis pathway, demonstrating significant whitening effects.

Further, in zebrafish larvae and embryo models, we evaluated the biological activity of lotus seed lily fermentation broth at safe concentration ranges of 2.5–10%, particularly its performance in antioxidant, whitening, and anti-inflammatory effects. The experimental results showed that the lotus seed lily fermentation broth significantly enhanced the total antioxidant capacity of zebrafish and demonstrated anti-inflammatory effects by inhibiting inflammatory cell migration, providing a scientific basis for its application in the field of anti-inflammation. Additionally, it exhibited strong whitening effects by strongly suppressing melanin synthesis at different concentrations. These findings provide a reliable foundation for elucidating the mechanisms of action of lotus seed lily fermentation broth and developing therapeutic applications.

The fermented liquid of lotus seeds and lilies not only showed multi-antioxidant, anti-inflammatory and whitening biological effects in cell and zebrafish models, but also showed significant inhibitory effect on *acne pathogenic bacteria*, providing experimental basis and scientific basis for its application as a natural antibacterial agent in acne treatment.

In summary, the fermented lotus seed and lily bulb extract possesses multiple biological activities such as antioxidant, whitening, anti-inflammatory, and antibacterial properties, with broad potential for functional applications. This research not only expands the application prospects of natural plant extracts in medical aesthetics and therapy but also highlights the potential of the concept of food and medicine sharing in modern medical research.

## Data Availability

The datasets presented in this study can be found in online repositories. The names of the repository/repositories and accession number(s) can be found in the article/supplementary material.
